# Point-of-Care Electroencephalography in Acute Neurological Care: A Narrative Review

**DOI:** 10.3390/neurolint17040048

**Published:** 2025-03-24

**Authors:** Roberto Fratangelo, Francesco Lolli, Maenia Scarpino, Antonello Grippo

**Affiliations:** 1 UOC Neurologia, Ospedale San Giuseppe, 50053 Empoli, Italy; 2 Department of Biomedical, Experimental and Clinical Sciences “Mario Serio”, University of Florence, 50134 Florence, Italy; francesco.lolli@unifi.it; 3 Neurophysiology Unit, Careggi University Hospital, 50134 Florence, Italy; scarpinom@aou-careggi.toscana.it (M.S.); antonello.grippo@unifi.it (A.G.)

**Keywords:** point-of-care EEG, portable EEG systems, rapid neurological diagnostics, non-convulsive status epilepticus management, traumatic brain injury assessment, stroke detection, delirium diagnosis, diagnostic accuracy, usability in healthcare, artificial intelligence

## Abstract

Point-of-care electroencephalography (POC-EEG) systems are rapid-access, reduced-montage devices designed to address the limitations of conventional EEG (conv-EEG), enabling faster neurophysiological assessment in acute settings. This review evaluates their clinical impact, diagnostic performance, and feasibility in non-convulsive status epilepticus (NCSE), traumatic brain injury (TBI), stroke, and delirium. A comprehensive search of Medline, Scopus, and Embase identified 69 studies assessing 15 devices. In suspected NCSE, POC-EEG facilitates rapid seizure detection and prompt diagnosis, making it particularly effective in time-sensitive and resource-limited settings. Its after-hours availability and telemedicine integration ensure continuous coverage. AI-assisted tools enhance interpretability and accessibility, enabling use by non-experts. Despite variability in accuracy, it supports triaging, improving management, treatment decisions and outcomes while reducing hospital stays, transfers, and costs. In TBI, POC-EEG-derived quantitative EEG (qEEG) indices reliably detect structural lesions, support triage, and minimize unnecessary CT scans. They also help assess concussion severity and predict recovery. For strokes, POC-EEG aids triage by detecting large vessel occlusions (LVOs) with high feasibility in hospital and prehospital settings. In delirium, spectral analysis and AI-assisted models enhance diagnostic accuracy, broadening its clinical applications. Although POC-EEG is a promising screening tool, challenges remain in diagnostic variability, technical limitations, and AI optimization, requiring further research.

## 1. Introduction

Point-of-care electroencephalography (POC-EEG) systems enable rapid brain activity assessment through reduced electrode arrays strategically placed at user- or manufacturer-specified locations [1,2]. These systems have extended the application of EEG beyond neurology departments into emergency departments (EDs), intensive care units (ICUs), and prehospital settings, serving both adult and pediatric populations [3,4,5,6].

POC-EEG was developed to address the logistical, technical, and interpretive limitations of conventional EEG (conv-EEG) for managing acute neurological conditions [7,8,9]. Among these, non-convulsive status epilepticus (NCSE), a common and potentially life-threatening condition in critically ill patients or those with acute brain injuries, requires rapid detection and intervention to reduce mortality and improve patient outcomes [10,11,12,13]. While continuous EEG (cEEG) remains the gold standard for detecting NCSE, according to the American Clinical Neurophysiology Society [14], its resource-intensive nature—requiring skilled technologists, neurologists, and substantial infrastructure—limits its feasibility in emergency and resource-constrained settings [15,16,17,18]. Initial studies on reformatted reduced-montage EEG (rm-EEG) have paved the way for streamlining EEG diagnostics [19,20,21,22,23,24,25,26,27].

Modern commercially available POC-EEG devices include fully prefabricated headbands and reusable and disposable EEG caps [28,29,30]. Advancements in array design, combined with the integration of advanced analytics and AI-driven algorithms, have significantly improved EEG diagnostics [7,9,31,32,33,34,35,36]. Furthermore, innovations such as simplified application methods, intuitive setups, and wireless data transmission have enhanced the accessibility of these devices [9,32,36,37]. These technological advancements are crucial for the democratization of EEG technology, expanding access to EEG not only for neurologists and neuroscientists but also for laboratory technicians, nurses, and frontline personnel in EDs and ICUs [38]. Beyond NCSE management, POC-EEG has expanded into diagnostic, therapeutic, and prognostic applications for traumatic brain injury (TBI), stroke, and delirium [1,2].

TBI is a significant public health concern, accounting for over 1.5 million ED visits annually in the U.S., with most cases classified as mild TBI (mTBI) or concussion [39]. Timely and accurate diagnosis of mTBI is critical, as delays can double recovery times and negatively affect cognitive, academic, and emotional functioning, with long-term risks of psychiatric disorders and reduced quality of life [40,41,42,43,44]. While current diagnostic tools often lack objectivity, EEG—and particularly quantitative EEG (qEEG)—has shown promise in detecting TBI by capturing changes in brain activity, such as shifts in power spectra, coherence, and phase synchrony [28,45,46,47,48,49,50,51,52,53]. Frontal EEG recordings, supported by diffusion tensor imaging (DTI) studies [54], have demonstrated high sensitivity for detecting mTBI, making POC-EEG systems practical and effective tools for bedside assessment [49,50,55,56,57].

Acute ischemic stroke (AIS) is a time-dependent condition that requires timely treatment to reduce morbidity and mortality [58,59,60]. However, current prehospital assessment methods, which are mainly based on clinical assessment scores, have some limitations with regard to diagnostic accuracy [61,62,63]. Processed EEG is a promising technique for early assessment of cerebral blood flow modifications, allowing key EEG biomarkers to be detected immediately after stroke onset and early identification of LVO stroke [64,65,66,67,68]. Several qEEG parameters are able to detect hypoxia-induced changes in brain electrical activity, allowing for the identification of early stroke-related alterations with high sensitivity and specificity [67,68,69,70,71,72]. The qEEG markers that have most successfully differentiated patients with AIS from healthy controls are the delta/alpha ratio (DAR) (signal delay) [69,73], the brain symmetry index (BSI) (hemispheric asymmetry) [72,74], and the reduced functional connectivity (lower phase synchronization) [73]. However, the ability of EEG to reliably distinguish between old and new lesions, ischemic and hemorrhagic strokes or stroke-like conditions, remains uncertain [75]. Some studies have analyzed the contribution of portable POC-EEG systems in the identification of LVOs and mimics [76]. These innovations could improve resource allocation and optimize the management of such patients, significantly reducing treatment delays [67,68,72,75,77,78,79].

Delirium, a common cause of altered mental status (AMS) in hospitalized patients, is associated with prolonged hospital stays, cognitive decline, institutionalization, and increased mortality [80,81,82,83]. However, it is frequently underdiagnosed due to the limitations of traditional clinical screening tools, which rely on subjective assessments and patient cooperation [84,85]. EEG provides an objective diagnostic alternative, with qEEG identifying characteristic EEG biomarkers of delirium [86,87]. POC-EEG systems may offer practical solutions for bedside delirium detection, enabling accurate diagnostics with minimal disruption to patient care [88,89,90].

This review explores the current evidence on the diagnostic reliability and operational feasibility of POC-EEG in the diagnosis of emergency neurological conditions, highlighting its applications across NCSE, TBI, stroke, and delirium and identifying areas for future research and technological advancement.

## 2. Materials and Methods

### 2.1. Protocol and Registration

A narrative review method [91] was adopted, involving three main steps: (1) a systematic search process with inclusion and exclusion criteria, (2) data extraction and synthesis of results, and an (3) analysis of key findings through narrative review. The protocol was registered in PROSPERO (CRD42024618850) before the study began.

### 2.2. Eligibility Criteria

The eligibility criteria for studies were defined considering the population, index test, and outcome relevant to the review question, with the reference test included when applicable. We included studies that evaluated the clinical performance, the diagnostic performance, and feasibility of POC-EEG systems, using conv-EEG as the reference standard when available. The study population consisted of participants who underwent POC-EEG for acute neurological conditions such as NCSE, TBI, stroke, and delirium. Original studies, including diagnostic cross-sectional and cohort studies, clinical studies, case-control studies, case series, and conference abstracts, were considered. Both comparative and non-comparative studies were included if they provided relevant data on the clinical performance, diagnostic accuracy, or feasibility of POC-EEG systems. Case reports were considered only if they provided unique insights into POC-EEG applications. Reviews, book chapters, opinion papers, and studies not available in English were excluded.

The outcomes assessed included the diagnostic performance of POC-EEG compared to conv-EEG, measured through sensitivity, specificity, predictive values, and agreement metrics. Additionally, the impact on clinical decision-making was evaluated, including whether POC-EEG influenced treatment choices, enabled earlier diagnoses, or improved patient outcomes such as mortality and neurological prognosis. The review also considered the effect on resource allocation, including reductions in hospital length of stay, avoidance of unnecessary treatments, and decreased inter-hospital transfers by enabling timely local decision-making. Furthermore, the feasibility and usability of POC-EEG were analyzed, focusing on ease of use by non-expert personnel, portability across different clinical settings, time efficiency in acquisition and interpretation, patient tolerability, signal quality compared to conv-EEG, and its role in optimizing the allocation of conv-EEG resources. Finally, studies assessing the integration of automated tools and artificial intelligence (AI) were considered with reference to their impact on the assessed outcome.

### 2.3. Search Process

A comprehensive search strategy was employed to identify studies published between 1 January 2001 and 20 November 2024. The search was conducted across Medline, Embase, Scopus, and ClinicalTrials.gov. The search strategy is detailed in the Supplementary Material. Automated searches were performed with predefined filters to exclude articles outside the relevant time frame, non-English publications, and studies containing irrelevant keywords.

**Figure 1:**
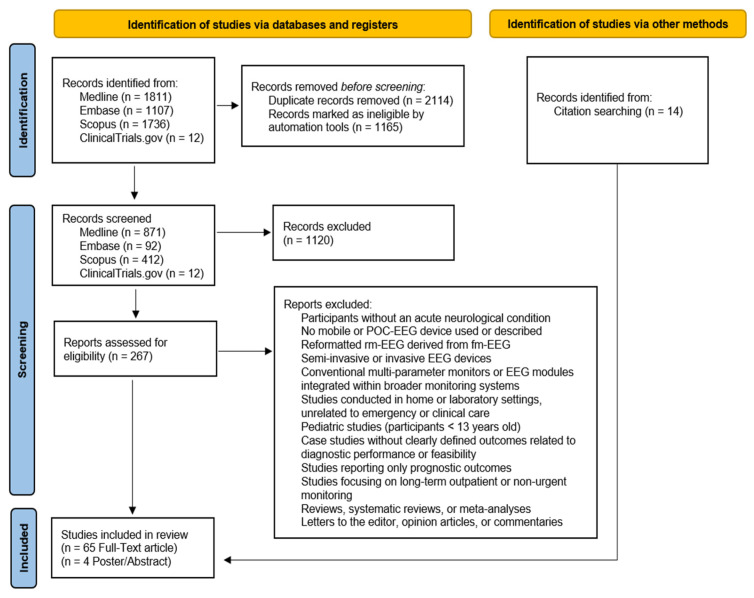
Flow diagram of the narrative review according to PRlSMA guidelines.

Identified records were imported into a reference management system, and duplicates were automatically removed. Semi-automated keyword-based screening reduced the initial dataset, followed by manual review of titles and abstracts. Subsequently, titles and abstracts were manually screened by two reviewers to assess initial eligibility, followed by a full-text review against the inclusion and exclusion criteria. The bibliographies of selected articles were reviewed to identify additional relevant studies. From an initial pool of 4653 records, 69 studies met the criteria for final inclusion. A PRISMA-adapted flow diagram [92,93] illustrating the search process is presented in Figure 1.

### 2.4. Data Extraction, Synthesis, Analysis, and Quality Appraisal

Data extraction was independently conducted by two authors using a predefined form, covering participant demographics, study design, device characteristics, and outcomes related to feasibility and diagnostic performance. Discrepancies were resolved through consensus or consultation with a third author. Due to variability in study designs and outcomes, statistical data synthesis and meta-analysis were not conducted. Instead, a descriptive synthesis was provided.

A formal quality assessment was not performed, aligning with the narrative review approach. However, study limitations—such as small sample sizes and potential biases—are discussed in the Limitations section to provide context for interpreting the results.

## 3. Results

Sixty-nine studies were included in this review, with the key information summarized across four tables categorized by clinical condition. Table 1 presents studies related to NCSE as it provides the most consistent data.

### 3.1. POC-EEG Systems in the Assessment of NCSE

#### 3.1.1. Evaluating Diagnostic Accuracy and Clinical Implications of POC-EEG Systems for NCSE Detection

The following section reviews 27 studies evaluating the use of POC-EEG in detecting NCSE in adult patient samples, emphasizing its diagnostic accuracy, clinical utility, and technological advancements across various clinical contexts. Of these, twenty focused on the Ceribell rapid-response EEG (rr-EEG) system, four investigated the CerebAir EEG headset, one study examined the Brainmaster EEG portable device, one evaluated the portable Grass Technologies Comet video EEG system, and one analyzed the Cadwell Ambulatory Monitor. Two studies specifically assessed post-cardiac arrest (PCA) patients, one focused on stroke and stroke mimic patients, while the remaining studies examined patients with suspected NCSE presenting with heterogeneous clinical conditions.

A preliminary study evaluated the Brainmaster portable EEG device (six electrodes) in 12 ED adult patients (median age 51.5) with AMS and seizure history or witnessed seizure, using non-continuous recordings (median 10 min) compared to conv-EEG. For all 12 patients, the POC-EEG interpretations by a remote neurophysiologist were identical to the conv-EEG interpretations by an on-call neurologist in determining whether the patient had NCSE or not. Only one patient (8%) was found to have a non-convulsive seizure (NCS). POC-EEG device cost was ~USD 2500 vs. ~USD 50,000 for standard EEG, suggesting potential cost-effectiveness for POC-EEG as a screening tool in the acute care setting [94].

Another study evaluated the Grass Technologies Comet (10 forehead electrodes) in 100 patients with unexplained AMS from various etiologies to rule out status epilepticus (SE). The system was compared to a full-montage (fm-EEG) in simultaneous 20-min recordings. Three expert neurophysiologists interpreted both EEGs blindly. Forehead EEG achieved a sensitivity of 50%, specificity of 100%, and accuracy of 98% for SE detection. No false positives were detected, but two of four SE cases were missed due to posterior brain activity beyond the recording area [95].

The CerebAir eight-electrode headset’s signal quality and diagnostic reliability have been evaluated across four studies, emphasizing its potential in critical care and emergency settings.

A study involving 50 neuro-ICU patients (median age 72) with AMS due to various etiologies (subarachnoid hemorrhage, cerebral hemorrhage, PCA syndrome, SE, and TBI) compared CerebAir monitoring with continuous conv-EEG (c-conv-EEG) for detecting abnormal EEG patterns, including periodic discharges (PDs), rhythmic delta activity (RDA), spikes and waves (SWs) and continuous slow waves (CSs) as well as for diagnosing NCSE. All EEG data were interpreted by one neurointensivist and one board-certified neurophysiologist. POC-EEG demonstrated a sensitivity of 97.4% and specificity of 90.9% for abnormal patterns, with PD detection showing 82.4% sensitivity and 97.0% specificity and CS showing 90% sensitivity and 97% specificity. For NCSE, sensitivity was lower at 70.6%, but specificity remained robust at 97.0%, confirming the device’s capability for accurate and rapid assessments in critical care environments [96].

A study involving 52 neuro-ICU patients (mean age: 63 years) with AMS due to various etiologies (e.g., repetitive epileptic seizures/SE, ischemic stroke, intracranial bleeding, meningitis, encephalitis, metabolic encephalopathies) compared CerebAir monitoring with a subsequent routine conv-EEG after a median delay of 24 h. Both were analyzed by a resident physician and supervised by a board-certified senior physician. The study revealed that CerebAir detected a higher rate of epileptiform discharges (34% vs. 15% with conv-EEG), with an overall agreement of 68% (*p* = 0.162). POC-EEG identified the same or additional ICU-relevant patterns (epileptiform discharges or seizures) in 89% of cases and showed 98% agreement in seizure detection. Background activity matched in 53% of cases (*p* = 0.126). CerebAir’s reduced channels effectively identified background activity and epileptiform discharges, demonstrating its adaptability and utility for long-term monitoring, although routine EEG (rEEG) followed after a median of 24 h, potentially affecting findings due to medication adjustments [98]. A separate study evaluated 40 ICU patients, including 20 from neurosurgical ICU with subarachnoid hemorrhage, cerebral parenchymal hemorrhage, or head injury (study group) and 20 from general ICU (control group). The cEEG was performed using the CerebAir headset in the study group and a simplified eight-electrode cEEG system in the control group. The EEG recordings were reviewed by an expert neurologist. EEG abnormalities were categorized into two groups: epileptic activity (EA), including generalized and focal seizures, SE, generalized PDs (GPDs), and lateralized PDs (LPDs), and non-EA, including focal or generalized slow-wave activity, sharp waves, and EEG asymmetries in frequency or amplitude. In the study group, 35% of patients exhibited EA, and 35% had non-EA, while in the control group, 25% exhibited EA and 45% had non-EA. However, these differences were not statistically significant (*p* > 0.05). POC-EEG monitoring led to the administration of anti-seizure medications (ASMs) in 50% of the CerebAir cases, compared to 35% in the control group [97].

Another study evaluated the CerebAir system in 100 neurological ED patients presenting with AMS or suspected seizures, with recordings lasting at least 10 min. Among 55 patients who underwent both POC-EEG and rEEG, POC-EEG was offline-interpreted by a neurology resident and supervised by an EEG expert, while rEEG was reviewed by specialized neurology residents under supervision of senior board-certified EEG experts. POC-EEG demonstrated 87.3% concordance in distinguishing normal, pathological slowing, or epileptiform activity. POC-EEG detected the same or additional pathological EEG patterns in 94.5% of cases, including interictal epileptiform discharges (IEDs), focal slowing, and generalized slowing. Among patients without immediate access to rEEG (9.3%), POC-EEG identified clinically significant findings, including IEDs and SE, which could have altered treatment decisions [99].

The signal quality and reliability of POC-EEG systems have been extensively evaluated across various settings. One study compared the performance of the Ceribell rapid-response EEG (rr-EEG) system to conv-EEG in two distinct environments: a controlled laboratory setting with a healthy subject and an ICU setting involving 22 patients presenting with AMS and suspected seizures. In ICU patients, EEG quality metrics (Hjorth parameters, artifact spike count, baseline wander, and kurtosis) showed no significant differences (*p* > 0.05) between rr-EEG and conv-EEG, except lower 60 Hz noise levels in rr-EEG due to its battery-operated design. In the laboratory, rr-EEG and conv-EEG showed visual and statistical agreement, with comparable correlation values [100].

Further insights into signal acquisition were provided by a subanalysis from the DECIDE trial, which examined seizures detected by both rr-EEG and conv-EEG in 19 patients. The study found that the electrographic features and laterality of seizures were comparable between the two systems. However, in cases where conv-EEG identified pathological activity that was missed by the rr-EEG, the abnormalities were not confined to the midline or parasagittal regions, supporting the conclusion that rr-EEG did not miss seizures due to reduced spatial coverage [103].

Several studies have demonstrated the effectiveness of rr-EEG in detecting seizures in critical care, with its technological innovations and its hub-and-spoke tele-EEG model, significantly improving care in resource-limited settings.

A study involving 38 patients with suspected NCSE from two ED sites assessed the diagnostic performance of the Brain Stethoscope, an auditory extension of the rr-EEG system that converts EEG signals into audible patterns to aid in seizure detection. Various etiologies were identified, including SE, stroke, TBI, toxic-metabolic encephalopathies, and idiopathic AMS. At the community hospital, emergency physicians independently interpreted rr-EEG using Brain Stethoscope. At the academic hospital, the on-call neurology fellow reviewed the first 15 min. At both sites, an attending neurologist remotely reviewed the full rr-EEG. Using sonified rr-EEG, emergency physicians at community hospital diagnosed two seizures—one confirmed as NCSE and one later reclassified as diffuse slowing—and ruled out seizures in 12 cases, achieving 100% sensitivity and 92% specificity in detecting seizures. Furthermore, emergency physicians reported a treatment change in six cases (43%) based on the sonified rr-EEG interpretation. At the academic hospital, rr-EEG identified ongoing seizures or HEP in three patients (11%) and NCSE in one patient (3%), leading to immediate ASM administration. Across both sites, rr-EEG led to diagnostic confirmation in 42% of cases, change in clinical management in 53%, and expedited patient disposition in 21% of cases [7].

The rr-EEG system was used in 10 patients with suspected SE, including cases of PCA, AMS, Central Nervous System (CNS) lesions, and epilepsy. The on-call neurologist received rr-EEG data through a cloud portal, interpreted it using sonification and visual review, and informed the attending physician of the SE diagnosis. Forty conv-EEGs were performed as needed, interpreted by a neurologist, and used as controls. The rr-EEG identified SE in 1/10 cases and ruled it out in 9/10 patients, reducing unnecessary medication use and contributed to palliative care decisions in two cases. In seven patients, conv-EEG followed rr-EEG showing concordance in six cases. The rr-EEG had one false-positive SE diagnosis, later ruled out by conv-EEG [27].

The DECIDE (Does Use of Rapid-Response EEG Impact Clinical Decision-Making) trial involved 181 patients with AMS across five U.S. hospitals and focused on the impact of rr-EEG on clinical decision-making. While awaiting conv-EEG, physicians—primarily neurology-trained with limited EEG experience—applied and interpreted rr-EEG bedside, assessing 30 s sonification per hemisphere and 60 s visual review on conv-EEG. The subsequent expert neurologists’ interpretations of the rr-EEG were used as the reference standard. The study aimed to compare the standard seizure management approach, where physicians rely solely on clinical judgment, with EEG-guided decision-making using rr-EEG raw data and EEG sounds. The rr-EEG identified seizures in 11% of patients, highly epileptiform patterns (HEPs) in 12%, and slow or normal activity in 78%. Compared to initial clinical suspicion, sensitivity for seizure diagnosis increased from 77.8% to 100% and specificity from 63.9% to 89%. The rr-EEG changed seizure suspicion in 40.2% of cases (*p* < 0.0001) and influenced treatment decisions in 20.1%. The rr-EEG increased confidence in diagnosis and treatment, with greater ICU experience further enhancing confidence in both assessments and decisions [9].

A study evaluated the rr-EEG system in 34 ICU patients (mean age 61 ± 18 years) with AMS (Glasgow Coma Scale [GCS] < 12) due to mixed etiologies (metabolic encephalopathy, AIS, intracerebral hemorrhage, TBI, and autoimmune encephalitis) and requiring EEG monitoring. Neurointensivists without epilepsy training listened to sonified EEG for 30 s per hemisphere and made treatment decisions before and after conv-EEG was used as a reference. Two epileptologists reviewed the rr-EEG recordings and correlated findings with conv-EEG reports. Compared to clinical suspicion, rr-EEG sonification improved NCSE detection sensitivity from 0 to 100% and increased specificity from 76% to 85%. Sonification alone influenced treatment decisions in 40% of cases, reducing unnecessary ASM use by 21–27%. However, it had limited spatial coverage and difficulty differentiating certain seizure-like patterns (artifacts, rhythmic movements, PDs), potentially leading to misinterpretations [37].

A study evaluated the effectiveness of the rr-EEG system sonification method in detecting seizures, comparing it to visual conv-EEG analysis. A total of 84 EEG samples were selected from patients with AMS, with patterns categorized by epileptologists into seizures, seizure-like abnormalities (e.g., GPDs, LPDs, triphasic waves, burst suppression), and non-seizure patterns such as slowing and normal activity. Medical students (*n* = 34) and nurses (*n* = 30) listened to sonified EEG clips, while neurologists (*n* = 12) and medical students (*n* = 29) visually reviewed the same recordings. All results were compared to the reference standard based on the majority consensus of three senior epileptologists. After brief training, nurses and medical students detected seizures by sound with high sensitivity (95–98%) and specificity (65–66%). They identified seizure/seizure-like events with 77% and 79% sensitivity and 82% and 85% specificity, respectively. Despite their elevated performance using audio EEG, students were less accurate with visual EEG, demonstrating 76% sensitivity and 65% specificity for seizures and 62% sensitivity and 65% specificity for seizures/seizure-like activity. EEG-trained neurologists analyzing visual EEG samples demonstrated 86% sensitivity and 87% specificity for seizure detection and 88% sensitivity and 90% specificity for seizures/seizure-like activity [31].

A study involving 10 neuro-ICU patients (mean age 59.7 years) with AMS at risk for NCSE showed that the rr-EEG system sound application facilitated decision-making. The rr-EEG recording lasted for up to 12 h. Data were interpreted on-site by treating physicians using real-time sonification and visual review on the rr-EEG device. Six patients underwent conv-EEG, interpreted by trained neurologists. The rr-EEG demonstrated 100% concordance (5/5 cases) with conv-EEG in ruling out seizures. The rr-EEG led to treatment modification in 40% of cases, with AED de-escalation in four of five high-seizure suspicion cases [32].

The rr-EEG system validation study assessed the Clarity AI-assisted seizure detection tool, which uses machine learning algorithms to provide continuous quantitative trends of seizure burden (SzB) and generate automated alerts when seizure activity exceeds predefined thresholds (≥10%, ≥50%, ≥90%). This multicenter evaluation across six hospitals analyzed 353 rr-EEG recordings from patients with AMS. The device transmitted data to a cloud server with expert neurologists categorizing the patterns into seizures, HEP (e.g., PDs or lateralized RDA), or normal/slowing activity. When tested against expert consensus, the Clarity algorithm at a 90% SzB threshold detected SE with 100% sensitivity and 93% specificity, with false-positive detection (6.8%) primarily due to HEP. SzB alarm thresholds of ≥50% and ≥10% had sensitivities of 100% and 88% and specificities of 82% and 60%, respectively. At 90% SzB, Clarity had 92.3% sensitivity for seizure episodes lasting more than 4.5 min. The overall sensitivity for seizure events of any duration was 88.2%, with a 99% negative predictive value (NPV) for seizure detection. The performance of the algorithm is considered reliable and valid at the extremes of the SzB spectrum—namely for SE and normal/diffusely slow activity—but less effective for detecting milder abnormalities [33].

In a U.S. community hospital, 317 rr-EEG recordings were analyzed post hoc using Clarity for SE detection, showing strong concordance with neurologist-confirmed SE cases and supporting its role in early detection and bedside patient care. Out of these, seven SE cases were identified by neurologists, while ten triggered a Clarity alert after 90% SzB threshold. Among the seven neurologist-confirmed SE cases, six had 100% SzB and one had 83.3% SzB. Four false-positive SE alerts were classified as seizure or HEP, indicating abnormal EEGs [34].

A retrospective study assessed Clarity’s accuracy in detecting SE using 665 POC-EEG recordings from 11 hospitals. Each EEG was reviewed post hoc by at least two blinded epileptologists (three in cases of disagreement) to establish a reference standard. The latest version of Clarity’s algorithm (v6.0) demonstrated 95% sensitivity, 97% specificity, 53% positive predictive value (PPV), and 99% NPV for SE detection at a ≥90% SzB threshold, improving from a prior version (95% sensitivity, 94% specificity, 32% PPV, 98% NPV). It missed one SE case (false negative) and generated 17 false positives, with only 1 misclassified as non-epileptiform. The accuracy of Clarity in detecting SE events underscores its potential to help clinicians rapidly identify, treat, or rule out SE in critical care settings [35]. A retrospective study of 235 deidentified rr-EEGs from the Yale New Haven Health System assessed the diagnostic accuracy of Clarity (version 6.0) in detecting HEPs (i.e., SWs, PDs, RDA, any pattern on the ictal–interictal continuum, brief potentially ictal rhythmic discharges, highly epileptiform bursts, or possible seizures), electrographic seizure (ESz), electrographic SE (ESE), or possible ESE (pESE), according to 2021 ACNS guidelines. Blinded expert neurophysiologists served as the reference standard. A >10% SzB threshold had high sensitivity (88%) for ESE/pESE detection with an NPV of 98%, making it effective for ruling out the condition. At >90%, specificity increased to 99% and PPV to 78%, supporting its diagnostic confirmation, though sensitivity decreased to 29%. A >10% threshold detected only 54% of ESz/ HEPs, with specificity of 88% and NPV 84%. Notably, 100% of definite ESz and 94% of ESz/HEPs were detected within the first 2 h, with 70% identified within the first 10 min [107].

A retrospective study conducted in a U.S. community hospital analyzed 157 ED adult patients (mean age 57.7) with unexplained encephalopathy, seizure-related events, or PCA, selected from 319 cases across the ED (49.2%), ICU (28.8%), and general floors (21.9%). The rr-EEG provided real-time automated seizure detection via Clarity AI, followed by expert neurologist review. POC-EEG identified seizures/SE in 14% of patients, HEP (including epileptiform discharges and rhythmic or periodic activity) in 21.7%, slow activity (including non-epileptiform burst suppression and generalized RDA) in 43.9%, and normal activity in 20.4%. A total of 51.6% of patients underwent conv-EEG later in their hospital stay, more often in epileptiform vs. non-epileptiform rr-EEG cases (69.6% vs. 41.6%). ASM treatment changed in 59.2% of cases after rr-EEG. A total of 50% of post-rr-EEG ASM-treated patients did not receive prior treatment, while 52.7% of pre-rr-EEG ASM-treated patients were no longer treated after rr-EEG. POC-EEG findings were significantly associated with ASM management changes (*p* < 0.001), leading to treatment escalation in 51.8% of patients with epileptiform activity (vs. 24.8% normal/slow activity, *p* < 0.001) and avoidance of unnecessary escalation in 26.7% of non-epileptiform cases (vs. 1.8% seizure/HEP, *p* < 0.001). ASM initiation was faster post-rr-EEG in seizure/HEP patients for both benzodiazepines and non-benzodiazepines. Longer hospital and ED length of stay (LOS) correlated with delayed POC-EEG initiation, while time to first ASM post-rr-EEG was not significantly linked to LOS. The study demonstrates that rr-EEG positively influences clinical decision-making, providing faster diagnoses and enabling more targeted treatment decisions [6].

An analysis of the rr-EEG system was conducted in two community hospitals, involving 88 ICU and ED patients (mean age 57) when conv-EEG was unavailable. A total of 19% exhibited hyperkinetic movements PCA, 46% had a history of seizures or witnessed convulsive activity, and 35% had AMS with concern for NCSE. The rr-EEG system recorded data for up to two hours, with an initial real-time Clarity-assisted interpretation performed by a critical care fellow. The Clarity algorithm used SzB alarm thresholds of <10%, 10–70%, or >70% to guide treatment decisions. A total of 21% had significant electrographic SzB, with 4% confirming on follow-up EEG and 5% requiring immediate transfer. POC-EEG ruled out or reduced concern for ESz in 78% of cases. After follow-up EEG, 2.4% were false negative. Compared to historical controls, transfers decreased from 2 to 1.1 per month, generating a net financial benefit of USD 13,936 per patient, covering fixed device costs [36].

In a study involving 74 ICU and ED patients with seizure-related events (49%), PCA (24%), and unexplained encephalopathy (27%), rr-EEG monitoring was integrated into a hub-and-spoke tele-EEG model. This system enabled EEG interpretation by transitioning from general neurologists at community hospitals to epileptologists at academic centers via a web portal. Transfer rates dropped from 44.6% (pre-rr-EEG) to 5% (post-rr-EEG, pre-tele-EEG) and 0% after tele-EEG implementation, preventing 94% of unnecessary transfers. The estimated cost savings from avoided transfers exceeded USD 39,000. These findings support rr-EEG combined with tele-EEG as an effective strategy to minimize transfers, optimize patient management in community hospitals, and reduce costs in resource-limited settings [8].

During a quality improvement (QI) project implementing rr-EEG, 164 EEGs were conducted for suspected seizures, including 35 conv-EEGs (pre-QI) and 115 rr-EEGs (post-QI). Diagnostic yield improved with rr-EEG, as 43% of rr-EEGs detected seizures or seizure-like activity, compared to only one instance of seizure-like activity with conv-EEG. Although the LOS was similar between groups, post-QI patients tended to have shorter LOS (from 7 to 4 days) with an annual cost-savings projection of approximately USD 737,818 due to reduced LOS, along with an additional ~USD 145,580 from diagnosis-related group (DRG) reimbursements [5].

The following studies evaluated the prognostic capabilities of POC-EEG systems, particularly in seizure risk prediction and clinical outcomes.

In a multicenter retrospective study of 240 patients (median age 64) with suspected seizure activity, 1 h rr-EEG was compared to follow-on conv-EEG for seizure risk prediction using the 2HELPS2B algorithm. Both rr-EEG and conv-EEG, the latter serving as the reference, were interpreted by EEG experts. The rr-EEG met non-inferiority to conv-EEG accuracy for seizure risk stratification, with comparable false-negative rates for seizure detection and survival analyses over 72 h. These findings suggest rr-EEG effectively triages patients, potentially reducing unnecessary prolonged conv-EEG for patients at low seizure risk, and optimizes resource allocation for higher-risk individuals [102].

A subanalysis from the SAFER-EEG (Seizure Assessment and Forecasting with Efficient Rapid-EEG) trial evaluated 283 ICU patients (221 rr-EEG, 62 conv-EEG) to compare diagnostic accuracy, clinical outcomes, and feasibility. POC-EEG significantly reduced ICU LOS (4.5 vs. 8.0 days, *p* = 0.003), particularly in patients with seizure or epileptiform activity, and was associated with better functional outcomes after adjusting for confounders (58% vs. 76% unfavorable modified Rankin Scale, *p* = 0.047). However, after matching for clinical variables, this association was no longer significant (*p* = 0.061). Mortality rates did not differ. Despite these advantages, the study’s retrospective design, potential selection bias, and unmeasured variables influencing clinical decision-making underscore the need for further prospective research to validate these findings [105].

The following studies specifically address NCSE detection in more specific populations, such as PCA and stroke patients.

A retrospective observational cohort study analyzed 70 patients who underwent rr-EEG monitoring following acute stroke evaluation. The cohort included 38 stroke patients (54.3%)—73.7% ischemic, 15.8% hemorrhagic, 10.5% TIA—and 32 stroke mimics (45.7%), with 46.9% seizures, 28.1% toxic-metabolic encephalopathy, and 12.5% hypertensive encephalopathy. EEG findings were categorized into seizure or SE, HEPs, slow activity, or normal activity. POC-EEG identified seizures or HEPs in six stroke patients (15.8%), including two found to be in ESE, and eleven stroke mimic patients (34.4%), two of whom were found to have focal electroclinical seizures characterized by expressive aphasia. It enabled rapid detection of epileptiform activity in stroke codes, aiding in stroke mimic differentiation without delaying acute stroke management. These findings support POC-EEG as a useful tool for detecting non-convulsive seizures in both stroke and stroke mimics [106].

One study assessed the Cadwell six-electrode POC-EEG system in 95 comatose PCA patients (mean age 59) in a tertiary cardiac arrest center. POC-EEG was applied for continuous monitoring (mean duration 354 min) until conv-EEG was initiated. Both were reviewed offline by an epileptologist and a neurointensivist on the first 30 min of recordings. The agreement between POC-EEG and cEEG findings was fair (kappa = 0.27). Seizures were detected in 2% of POC-EEG cases, compared to 4% with c-conv-EEG. Neither POC-EEG nor cEEG findings were significantly associated with survival outcome [29].

A study on 21 comatose PCA patients (median age 64) evaluated the utility and limitations of the Clarity algorithm (version 4.0) for automated seizure detection. The rr-EEG analysis by board-certified epileptologists identified ESz in 19% of patients, with ESE in 9.5%. However, the Clarity system failed to detect any of these abnormalities, exposing its limitations in sensitivity for seizure detection in PCA patients and underscoring the need for expert review. Moreover, the system did not account for other EEG abnormalities such as isolated epileptiform discharges, rhythmic or periodic patterns, or brief potentially ictal rhythmic activity (RA) [104].

#### 3.1.2. Evaluating Feasibility of POC-EEG Systems for NCSE Detection

Here is a summary of 21 studies on the feasibility of POC-EEG systems for evaluating NCSE or AMS in various clinical settings.

The Brainmaster six-electrode portable EEG device tested in ED patients was easy to set up by an EEG technologist, with a median time from consent to EEG of 10 min and 38 min from EEG ordering to interpretation (vs. 3 h for conv-EEG). A wired Internet port was necessary to allow real-time remote access to recordings [94].

The Grass Technologies Comet EEG system was adaptable to emergency situations but reported 2% unsatisfactory recordings due to artifacts [95].

A study involving 10 patients with suspected SE demonstrated the feasibility of the rr-EEG system. The study included 40 control patients who underwent conv-EEG for comparison. The rr-EEG was applied by nurses, respiratory therapists, or EEG technologists with an average setup time of less than 10 min. The mean time to interpretation was 23.8 min, significantly faster than conv-EEG, which took 126.5 min. No on-call EEG technologist was required for rr-EEG, while 15/40 conv-EEG cases required after-hours call (*p* = 0.02). The rr-EEG was successfully deployed in various settings, including respiratory isolation rooms and prone-position patients, with staff providing positive feedback on its usability. Minor impedance issues were resolved with adjustments [27].

A separate study of 38 participants across two hospitals highlighted the efficiency of the rr-EEG system in EDs, with a median time from EEG start to diagnosis of 75 min, showcasing its effectiveness in time-sensitive situations [7].

In the DECIDE trial, the rr-EEG system showed high feasibility and portability. The system enabled Rapid-EEG data acquisition, with a median setup time of 5 min, with physicians setting up and using it without EEG technologists. Physicians rated the device and headband setup as easy. Additionally, the rr-EEG system facilitated rapid data acquisition, ensuring minimal disruption to clinical workflows in ICU settings. The system’s portability was evident, with electrodes easily applied and wireless data transmission to a cloud server for remote review. Safety and tolerability assessments revealed no serious adverse events, with only 1 of 181 patients experiencing scalp irritation [9].

In another prospective study involving 34 ICU patients, the rr-EEG system was used for rapid bedside recordings. The headband was placed by attending physicians or neurology fellows after a 4 min training video, eliminating the need for trained EEG technologists. Setup time was quick (6 ± 3 min). Time-to-EEG was significantly faster than conv-EEG (median 23 min vs. 145 min, *p* < 0.001). It was rated easy to use by physicians. The device transmitted data wirelessly for remote review, allowing use in various clinical settings. Signal quality was sufficient for seizure detection, but one-channel sonification limited detailed EEG analysis [37].

The rr-EEG sonification method was tested on 34 ED patients with AMS using single-channel temporal recordings converted to sound. After a brief 4 min instructional video, it enabled rapid interpretation but had limited spatial coverage [31].

A study evaluated the feasibility of rr-EEG in a community hospital ICU where conv-EEG was not available 24/7. It was applied by ICU staff, who rated it easy to use, with a mean setup time of 5.0 ± 2.4 min, significantly faster than conv-EEG (*p* < 0.001). The system provided real-time sonification and on-device visual EEG display, with data transmitted via Wi-Fi for remote review [32].

A study on 235 rr-EEG recordings in critically ill patients undergoing monitoring found a median duration of 3.6 h, though 29% became uninterpretable due to signal degradation and artifact, typically after 2 h 23 min [107].

In a cohort of 157 ED patients, the rr-EEG system was used for rapid monitoring, with a median duration of 2.1 h. Median door-to-EEG time was 1.2 h. Notably, 55% of studies were conducted after hours, showing high feasibility in time-sensitive settings [6].

Similarly, in a study involving 88 ICU and ED patients, the rr-EEG system was successfully used by critical care fellows to monitor patients suspected of having seizures or encephalopathy, with rapid setup and no delays in patient care [36].

Another study involving 74 patients across ICU and ED settings found that 69% of POC-EEG studies were performed after hours when conv-EEG was unavailable. A tele-EEG hub-and-spoke model enabled remote epileptologist interpretation [8].

In a study conducted as a QI project at a community hospital, the protocol enabled rr-EEG initiation by nurses, physicians, and advanced practice providers in ED and ICU settings. The median time-to-EEG for POC-EEGs was significantly shorter (74 min) compared to conv-EEGs (562 min). The use of POC-EEG increased hospital EEG volume 3.29 times, especially in ED [5].

In the SAFER-EEG subanalysis, the rr-EEG system was implemented in 283 ICU patients across four institutions. POC-EEG was initiated by various healthcare providers in 66% of cases, compared to 31% for conv-EEG (*p* < 0.001). The POC-EEG cohort had significantly faster door-to-EEG times (median 6.1 h) compared to conv-EEG (median 25.3 h, *p* < 0.0001) [105].

A retrospective study of 70 patients who received POC-EEG after acute stroke evaluation revealed that the procedure was seamlessly integrated into clinical care, providing real-time diagnostic insights without delaying stroke management [106].

A retrospective case series of five ICU patients with COVID-19 showed that the rr-EEG system was effectively used for monitoring. Setup was quick, performed by nursing staff, and provided timely diagnostics. The conv-EEG was used for confirmation in two patients [101].

CerebAir wireless system, a portable eight-electrode headband with Bluetooth transmission to a bedside EEG computer, was used for continuous recording in the ICU. While recordings were planned for 22.2 ± 9.5 h, only 15.5 ± 8.9 h were analyzable due to electrode detachment and impedance issues [98].

In one neurocritical care study, CerebAir operated cEEG monitoring. The median time-to-EEG was 57 min, representing a reduction of 303 min compared to c-conv-EEG. The median monitoring duration was 134.5 min, and no adverse events were reported, highlighting its feasibility for prolonged use. It was equipped with a video camera and Bluetooth transmission to allow EEG data transmission to a conventional computer [96].

In a study of 40 ICU patients, CerebAir was applied by ICU physicians without EEG technicians, while a simplified eight-electrode EEG was applied by an EEG technician. CerebAir enabled quicker electrode setup (6.2 ± 1.1 min vs. 10.4 ± 2.3 min, *p* < 0.0001) and allowed recordings exceeding 24 h in 43% of cases. However, it required more artifact corrections than conv-EEG (35 vs. 11 interventions, *p* < 0.01), and pressure lesions were observed in 17 patients, leading to monitoring interruption in four cases (~52 h) to prevent further skin damage [97].

Additionally, in a cohort of 100 neurological ED patients, CerebAir was set up in a median time of 7 min by non-specialized staff. Application was challenging in patients with long/thick hair or lack of cooperation. Data were transmitted via Bluetooth for offline review. The data were available outside regular hours, including evenings and weekends, ensuring EEG access when rEEG was unavailable. Impedance issues, especially in the occipital electrodes, sometimes affected signal quality [99].

The Cadwell Ambulatory Monitor (six electrodes) was tested in PCA ICU patients. Setup was easy and rapid for non-specialists. Continuous recording lasted until conv-EEG was initiated. Due to signal artifacts from electrical interference, it was recorded in 100% of cases but was interpretable in 60%, while conv-EEG was interpretable in 87% [29].

### 3.2. POC-EEG Systems in the Assessment of TBI

#### 3.2.1. Evaluating Diagnostic Accuracy and Clinical Implications of POC-EEG Systems for TBI Evaluation

POC-EEG devices have been increasingly analyzed in twenty-eight studies for their clinical impact and diagnostic performance in TBI assessment. Of these, twenty-five focused on the BrainScope portable EEG device, one analyzed the Lifeware Labs device, one evaluated the Ceribell rr-EEG system, and one examined the Covidien BIS-monitor.

A foundational study evaluated 153 ED patients presenting with AMS or headache using the BrainScope device. The EEG system recorded frontal brain activity, processed it with advanced algorithms to generate a Brain Abnormality Index (BAI), and classified results as normal or abnormal. Disorders included intracerebral hemorrhages (22), strokes (11), tumors (9), and concussions (14). The BAI demonstrated a sensitivity of 96% and specificity of 87% for detecting abnormalities, with scores correlating to disease severity, highlighting its potential for rapid neurological triage, especially concussions [108].

Another study validated a qEEG algorithm in 105 patients with mTBI, including 53 brain Computed Tomography-positive (CT+) and 52 Computed Tomography-negative (CT−) cases, compared to 50 ED controls. POC-EEG recorded 10 min of resting EEG. The TBI discriminant score (TBI-DS) achieved 92% sensitivity for functional impairment in CT+ patients while 34.6% of CT− cases were also classified as brain-injured, suggesting possible concussion-related brain dysfunction despite normal CT [109].

Similarly, a study validated a qEEG algorithm initially developed in EEG research on 396 college athletes. Among this population, 28 athletes who sustained mTBI were tested at injury onset, day 8 and day 45 post-injury. By day 8, neuropsychological testing results had returned to baseline, but the qEEG findings remained abnormal through day 8 (*p* < 0.05), resolving by day 45, particularly in coherence (decreased), asymmetries (increased), and power (increased beta-band activity). It was only by day 45 that qEEG measures returned to baseline values. Findings suggest qEEG detects prolonged physiological recovery beyond symptom resolution [110].

Using the same index and methodology, a study on 65 mTBI athletes evaluated within 24 h of concussion, with follow-ups at 8 and 45 days, reported that the TBI Index demonstrated greater elevation in moderate cases across all time points compared to mild cases. A higher TBI Index at injury was associated with delayed return-to-play (RTP) outcomes, unlike symptom or cognitive assessments, which normalized by day 8 and failed to predict RTP duration [56].

Similarly, another study assessed concussion recovery in 59 injured athletes compared to 31 controls using the mTBI discriminant score (MTBI-DS) algorithm to classify brain dysfunction based on previously established EEG features. While specific details about the discriminant function were not provided, POC-EEG data and clinical assessments were collected at injury onset, day 8, and day 45 post-injury. Concussed athletes had higher post-concussive symptoms (*p* < 0.05) and poorer cognitive scores (*p* < 0.05) at injury, normalizing by day 8. They showed significant EEG abnormalities persisted until day 8 and resolving by day 45, despite normalized clinical assessments [111].

Another study also assessed the utility of the TBI Index discriminant function, though it did not include a control group. The study compared the TBI Index classification outcome with medical examination and CT scan findings. A total of 119 ED patients with mTBI were categorized into CT+ and CT− groups, with the majority evaluated within 12 h from injury. The TBI Index achieved high sensitivity (94.7%) in discriminating between CT+ and CT− groups, with specificity at 49.4%, a PPV of 47.4%, and an NPV of 95.3%. Notably, the specificity was more than double that observed using the New Orleans Criteria (NOC) in such populations (23.5%). Moreover, when combined with the NOC, the sensitivity of the classification system increased to 97.4%, while specificity remained at 50.6%. Findings suggest EEG-based screening may reduce unnecessary CT scans [57].

A study involving 499 ED patients developed a sequence of binary classifiers using machine learning-based qEEG features from POC-EEG data to classify TBI patients into three categories: normal, concussed, and CT+ structural injury. The classification process sequentially applied binary discriminant functions, each incorporating multiple EEG variables (e.g., power, coherence, phase synchrony, fractal measures). The approach aimed to improve detection accuracy and differentiation between mild and severe forms of TBIs. The classifier distinguishing CT+ cases from other conditions achieved 96% sensitivity and 78% specificity, with an NPV of 99%. Performance for concussion detection showed 80% sensitivity and 74% specificity. These results suggest that the BrainScope discriminant function effectively detects mTBI, differentiating it from structural brain injuries (CT+ cases) and reducing the incidence of false-negative classifications [50].

Additionally, a study aimed to develop and validate a machine learning-based classifier using BrainScope’s handheld EEG device to differentiate CT+ structural brain injuries from CT– and non-TBI cases in emergency settings. The study enrolled 1470 participants across 16 U.S. EDs including individuals with mild-to-moderate concussions, normal controls, and those with CT+ structural brain injuries. The classifier analyzed qEEG features including power spectra, frequency bands, and connectivity measures. Three different classifier methodologies—Ensemble Harmony, Least Absolute Shrinkage and Selection Operator (LASSO), and genetic algorithm (GA)—were tested. All classifiers demonstrated high sensitivity (97.5%) and moderate specificity (around 59.5%) for CT+ cases. Two classifiers showed 100% sensitivity for clinically significant injuries like traumatic hematomas. The EEG-based classifier demonstrated an NPV above 99% and a PPV of approximately 26%. BrainScope’s classifier demonstrated superior specificity compared to existing clinical guidelines, highlighting its potential to reduce unnecessary CT scans. The study concluded that this portable EEG device is feasible for rapid TBI assessment, aiding in early triage decisions [112].

In a study involving 38 adults with CT+ hematomas and 38 CT− controls, the TBI Index achieved 100% sensitivity, 66% specificity, a 74.5% PPV, and 100% NPV, correlating significantly with hematoma volume but not bleed type or distance from electrodes. Findings suggest the TBI Index effectively detects intracranial hematomas and may aid in acute TBI assessment [28].

Prospective evaluations of intracranial hematomas using the TBI Index in 116 CT+ patients (46 with hematomas) and 278 CT− patients demonstrated a sensitivity of 95.7% and specificity of 43.9% for detecting hematomas. The analysis showed two false negatives (CT+ misclassified as CT−) and 156 false positives (CT− misclassified as CT+), reflecting the trade-off in prioritizing sensitivity over specificity. Most hematomas were classified as Marshall Score II, with a mean blood volume of 16.4 cc. Sensitivity was unaffected by hematoma volume or distance from electrodes, confirming the robustness of the TBI Index across different hematoma characteristics [113].

A prospective observational study compared a handheld qEEG device to clinical decision rules (NOC, Canadian CT Head Rule [CCHR], and National Emergency X-Radiography Utilization Study [NEXUS] II) in predicting intracranial CT lesions in 152 patients presenting to two urban EDs with acute mTBI. Using 10 min of frontal lead data, a qEEG discriminant score (cutoff ≥31) demonstrated high diagnostic performance for CT+ cases (AUC = 0.84; 95% CI, 0.76–0.93), achieving 92.3% sensitivity and 57.1% specificity, with three false negatives and 54 false positives. Compared to clinical decision rules, qEEG had higher specificity than NOC (15.8%) and NEXUS II (31.7%) while maintaining comparable sensitivity (>90%). CCHR had better specificity (86.5%) but significantly lower sensitivity (46.1%). These findings highlight qEEG’s potential as a rapid and effective tool for TBI triage in the ED [114].

An observational, multisite, prospective clinical trial involving 720 ED adults with mild-to-moderate TBIs assessed the diagnostic accuracy of EEG-based technology for detecting structural brain injuries. The BrainScope device, utilizing the Structural Injury Classifier (SIC) in binary classifications, demonstrated a sensitivity of 92.3% for CT+ cases, including hematomas >1 mL, and a specificity of 51.5%, with 12 false negatives and 273 false positives. When using a ternary classification that included an equivocal category, sensitivity increased to 97.4%, though specificity dropped to 38.7%. The integration of EEG features with clinical signs improved classification performance, achieving an AUC of 0.82 compared to 0.75 for clinical data alone. Findings suggest EEG-based classification enhances CT triage and my contribute to reducing unnecessary scans, particularly in patients with minor or no functional impairment [49].

The Brain Function Index (BFI), derived from qEEG recordings obtained using the portable BrainScope device, was evaluated as an objective marker for functional brain changes in a study of 296 CT—subjects aged 18–40. Significant differences in BFI were observed between asymptomatic controls and mTBI patients (*p* = 0.045), with greater impairment in those with moderate mTBI (*p* = 0.012), highlighting its potential for early detection of functional brain changes in CT− mTBI cases, aiding in early detection and clinical management [115].

In a separate study of 713 adults presenting to EDs with closed head injuries (GCS scores 12–15), the BrainScope Ahead 300 POC-EEG was evaluated for its ability to detect functional brain impairment. The EEG-based BFI showed a significant correlation with functional impairment severity (*p* < 0.0001) and demonstrated a clear distinction between CT+ patients (structural injury) and CT− mTBI patients. Findings suggest BFI scales with functional impairment and may aid early mTBI assessment in CT-negative patients [48].

To establish normative cognitive data for the BrainScope Ahead 300 device, 624 participants aged 18–80 were recruited from diverse U.S. regions. The portable EEG device, coupled with cognitive tests assessing reaction time and processing speed, produced results consistent with PC-platform norms. Age-related declines in cognitive throughput were noted (*p* < 0.0001), while no significant gender effects were found. These findings support the reliability of these tests for assessing mTBI in clinical and community settings [116].

A study evaluated 719 adults (ages 18–85, GCS 13–15) using BrainScope One, an EEG-based classifier incorporating 5–10 min frontal EEG and clinical risk factors. It achieved 92% sensitivity, 98% NPV, AUC 0.82, and reduced CT referrals by 26% (564 to 416) compared to standard clinical practice. This led to a 33% decrease in false positives (408 to 272), lowering the false discovery rate from 72% to 65% (*p* = 0.01). The false-negative proportion was 7.7%, but no missed cases required neurosurgery or showed symptom exacerbation. These findings suggest the device’s utility in minimizing unnecessary CT scans without compromising clinical safety [47].

In another study, 354 male high school and college contact sport athletes (244 controls and 110 concussed) were evaluated using BrainScope Ahead devices. The BFIs calculated from qEEG data were significantly lower in concussed athletes within 72 h of injury compared to controls (*p* = 0.0036), with no significant differences observed at 45 days post-injury, supporting the BFI’s utility in tracking concussion recovery [55].

A study involving 364 (ages 13–25) athletes (177 concussed, 187 controls) from 42 U.S. sites assessed participants using the BrainScope Ahead Concussion Assessment System at multiple time points (day 0, day 5, RTP, and RTP + 45). The enhanced BFI (eBFI) was calculated using a multimodal classifier integrating EEG features, neurocognitive performance, and vestibular symptoms. Significant differences in eBFI were observed between injured and control groups at day 0 and day 5, indicating measurable brain function impairments in concussed athletes. Prolonged recovery group (RTP ≥ 14 days) had persistently lower eBFI scores compared to short recovery group (RTP < 14 days), validating the system’s utility for concussion monitoring [117].

In a study of 91 patients with closed head injuries, the EEG-based SIC was used to guide CT referrals. After data acquisition with the BrainScope One device, a real-time embedded machine learning algorithm processed selected EEG data and clinical symptoms to generate the SIC output. The SIC recommended CT scans for only 63 patients, compared to 91 based on clinical judgment, resulting in a 30.8% reduction in CT referrals. Sensitivity was 100%, with no false negatives, confirming that all 13 CT+ cases were correctly identified. Notably, the SIC correctly identified one case as an old infarct rather than an acute injury. These results highlight the SIC’s ability to reduce unnecessary CT scans and minimize radiation exposure [118].

In a retrospective study, an EEG-based algorithm assessed the likelihood of TBI in 701 patients presenting to the ED within 72 h of a closed head injury, with or without the influence of drugs and alcohol (DA). The SIC, an EEG-based algorithm incorporating select clinical features (e.g., Loss of Consciousness and AMS), demonstrated 91.8% sensitivity and 52.3% specificity for the entire group. No significant difference in sensitivity was found between patients with and without DA, but specificity was significantly lower in the DA group (29.3% vs. 56.2%, *p* < 0.0001), suggesting that consciousness alterations may act as “TBI mimics” in intoxicated patients. Integrating SIC biomarkers into CT clinical triage reduced unnecessary CT scans by approximately 30% in DA-positive patients compared to traditional clinical practice, enhancing triage accuracy especially in patients under the influence of substances [119].

Another study involved 31 collegiate athletes aged 17–24, including 18 with sports-related concussions (SRCs), using the BrainScope EEG system to collect 5–10 min of EEG data. The BFI was computed using EEG, cognitive, and vestibular data. Results demonstrated significant correlations between DTI metrics and BFI, especially in the corpus callosum and inferior fronto-occipital fasciculus. Higher BFI scores were associated with changes in white matter diffusivity, highlighting functional impairment in concussed athletes [54].

A study of 186 high school and college athletes (87 concussed, mean age 18.1; 99 non-injured controls, mean age 17.8) employed the portable EEG device BrainScope Ahead 300 iP device to collect 5–10 min of EEG data. The eBFI was computed from a combination of EEG, neurocognitive, and vestibular/balance features. Concussed athletes had significantly lower eBFI scores initially and at day 0 (injury) and day 5 (*p* < 0.001), which normalized by RTP and 45 days post-RTP (*p* > 0.8). Sex differences were observed in symptom severity but not in eBFI scores [120].

Another investigation evaluated 580 (ages 13–25) high school and collegiate athletes (207 concussed, 373 controls) across 10 U.S. sites using the Concussion Index (CI), a machine learning-based, multimodal tool that combines EEG features, neurocognitive tests, and clinical symptoms. At injury, concussed athletes showed significantly lower CI scores (mean 32.7) than controls (mean 75.0), demonstrating high sensitivity (86%) and specificity (70.8%), with an NPV of 90.1% and a PPV of 62%. It strongly correlated with symptom severity (R^2^ = 0.64). Athletes with prolonged recovery (RTP ≥14 days) had lower initial CI scores than those with rapid recovery. By RTP, 78.2% of concussed athletes had normalized CI scores, supporting its utility in objectively diagnosing concussions and recovery monitoring, aiding RTP decisions [121].

A study analyzed 771 participants (aged 13–70) with concussions, assessed within 120 h using a portable EEG device. The qEEG was recorded, and subtypes were classified. Five electrophysiological concussion subtypes were classified, with Subtypes 2 and 4 associated with prolonged recovery and Subtypes 3 and 5 with rapid recovery. The machine learning classifier distinguished subtypes with 96.6% classification accuracy, demonstrating qEEG’s potential for personalized concussion management and recovery predictions [122].

Another study evaluated the utility of bispectral index (BIS) monitoring to diagnose brain death (BD) in patients with severe TBIs. The Covidien BIS-monitor system used four forehead electrodes to continuously record EEG signals, which were processed by a computer-based proprietary algorithm to generate a BIS score (0–100). Among 62 ICU patients with severe TBIs, BIS values showed a significant correlation with admission GCS (r = 0.43, *p* = 0.001). The system effectively identified patients with BD (BIS = 0) and differentiated them from survivors (mean BIS = 39.2 ± 9.0), demonstrating its feasibility and clinical utility for continuous monitoring and prognostication in severe TBI cases [30].

Recent advancements in prehospital EEG technology have improved TBI management in austere settings. A retrospective evaluation of 13 U.S. soldiers with closed head injuries in military settings utilized the BrainScope One portable EEG device within 72 h post-injury. The SIC algorithm, generated in real-time by the device, categorized patients as Negative (no structural injury), Positive (likely structural injury), or Equivocal (requires further evaluation). It demonstrated a 100% NPV, correctly indicating no need for CT scans in 11 of the 13 cases, with 1 false-positive case. This highlights the device’s potential to aid medical decision-making and reduce unnecessary imaging in austere environments [123].

In a pilot study assessing the rr-EEG system during Critical Care Air Transport (CCAT) training, the device proved effective even in a simulated transport environment. The team successfully navigated the logistical challenges of aircraft noise and vibrations, setting up the device swiftly and correctly identifying simulated NCS/NCSE patterns through both visual and sonified EEGs. These findings underscore the feasibility of rr-EEG for real-time seizure detection during aeromedical transport [124].

A prospective pilot study recorded single-channel EEG data (Lifeware Labs Apollo device) from 40 trauma patients during air ambulance transport. EEG was processed offline using computer algorithms, selecting 90 s artifact-free segments for frequency analysis, focusing on delta, theta, and alpha power. Notable findings included an inverse correlation between EEG delta power and Ubiquitin C-terminal hydrolase L1 (UCH-L1) biomarker levels, although no significant differences were observed between CT+ and CT-groups. The results affirm the feasibility of acquiring reliable EEG data in prehospital settings, offering potential applications for early TBI management [125].

#### 3.2.2. Evaluating Feasibility of POC-EEG Systems for TBI Evaluation

We identified 15 studies about the feasibility of POC-EEG devices in assessing TBIs.

The BrainScope device, employing a frontal electrode montage (5–10 electrodes) and automated artifact removal algorithms, was extensively tested in ED environments.

In one study involving 153 ED patients with AMS, the BrainScope demonstrated a setup time under five minutes and provided robust signal quality and automated processing, with healthcare professionals reporting ease of application and minimal training requirements [108].

Similarly, in a study with 105 ED patients with mTBI, the device was highlighted for its portability, minimal operator expertise requirements, and artifact rejection capabilities [109].

A study on 28 concussed athletes confirmed excellent portability and signal quality, with data collection feasible at injury onset and during recovery [110].

Another study involving 65 concussed athletes reinforced its ease of use and rapid setup, with health professionals reporting high satisfaction with its usability [56].

Additionally, 59 concussed athletes demonstrated prolonged signal quality for concussion assessment, even when clinical symptoms normalized, highlighting the device’s reliability for tracking recovery [111].

In collegiate settings, a study on 110 concussed athletes demonstrated rapid electrode application and high signal accuracy, validating its use in tracking symptom resolution and RTP decisions [55].

In ED-based studies, BrainScope has proven effective in diverse clinical scenarios. A study of 119 patients classified as CT+ or CT− underscored its rapid application time, automated processing, and real-time analysis compatible with CT imaging [57].

In another study of 38 CT+ hematoma patients and 38 CT- controls, the device demonstrated consistent signal quality with electrode placement completed in under five minutes [28].

A separate study with 46 CT+ and 278 CT− mTBI patients reported quick application times and portability, solidifying its role in ED triage workflows [113].

Another investigation involving 152 mTBI patients demonstrated the device’s fast setup and portability in the ED, enhancing diagnostic workflows [114].

In 719 ED patients (GCS 13–15, ages 18–85) with mTBI, BrainScope One demonstrated feasibility as a portable, rapid EEG-based tool with setup <5 min and automated artifact rejection, making it suitable for efficient use in emergency settings [47].

In 91 ED patients (ages 18–76, GCS 14–15, ≤3 days post-injury), the portable BrainScope One device recorded 5–10 min of EEG with setup <5 min. It was operated by physician assistants, nurses, and technicians, demonstrating clinical usability [118].

The BrainScope has also shown feasibility in military and transport settings. A study on 13 U.S. soldiers with TBIs confirmed its portability and signal integrity, making it suitable for austere conditions [123].

The rr-EEG system, tested in simulated aeromedical transport settings, proved effective under noisy and vibration-prone conditions, enabling swift setup and accurate seizure detection [122].

Similarly, the Lifeware Labs device, used in air ambulances with trauma patients, achieved successful single-channel recordings in 84% of cases, demonstrating feasibility in prehospital environments [125].

### 3.3. POC-EEG Systems in the Detection and Management of Strokes

#### 3.3.1. Evaluating the Diagnostic Accuracy and Clinical Implications of POC-EEG Systems in Stroke Assessment

We identified six studies highlighting the diagnostic potential and clinical implications of POC-EEG devices in stroke assessment. Among these, two examined the Muse portable EEG device, two assessed the Waveguard Touch EEG system, one evaluated the BrainLab EEG recorder, and one analyzed the BrainScope EEG device.

In a prospective study, the BrainLab EEG recorder was used to acquire raw EEG data from 21 patients with acute unilateral supratentorial strokes within 24 h of admission, using an eight-channel setup. The EEG data were then processed offline using Matlab software to calculate the BSI, which quantifies hemispheric asymmetry. The results revealed a strong correlation between BSI and NIHSS scores (r = 0.86, *p* < 0.01), with a BSI of approximately 0.18 for severe strokes and 0.06 for mild strokes [72].

A retrospective study used the BrainScope system to process EEG data from 48 stroke patients (31 ischemic, 17 hemorrhagic) and 135 stroke mimics. Raw EEG from frontal electrodes was acquired and processed offline using an automated algorithm to compute the Structural Brain Injury Index (SBII). The system achieved 91.7% sensitivity and 50.4% specificity for stroke detection, with 94.4% NPV and 39.6% PPV. False positives accounted for 49.6% of stroke mimic classifications, reducing specificity. False negatives (8.3%) were mainly in cerebellum, pons, and thalamus. Additionally, it correctly identified 80% of ischemic strokes that were initially missed on CT but later confirmed by MRI. These findings suggest that POC-EEG may provide rapid, diagnostic-ready outputs from raw EEG data for stroke triage [77].

A study evaluated the four-channel Muse EEG device in 33 ischemic stroke patients and 25 healthy controls. EEG was recorded within 48 h of stroke onset, with raw data transmitted via Bluetooth for offline processing using Matlab software. The Revised BSI (rsBSI) was calculated from spectral power asymmetry between hemispheres. The rsBSI demonstrated significant differentiation between stroke patients (median rsBSI = 0.363) and controls (median = 0.1686, *p* < 0.05). Higher rsBSI correlated with NIHSS scores, indicating greater neurological impairment. Follow-up rsBSI values decreased after 2–3 months (median = 0.213, *p* = 0.002), indicating recovery. These results highlight the feasibility of using the Muse device for stroke monitoring, although requiring post-acquisition analysis [74].

A further study used the Muse portable EEG device to record 3 min EEG data from 16 ischemic stroke patients and nine controls. Data were analyzed offline using wavelet transforms in Matlab. The Pairwise-derived BSI (pdBSI), DAR, and (Delta + Theta)/(Alpha + Beta) Ratio (DBATR) were calculated. These values were used for classification analysis with a random forest algorithm (TreeBagger). In stroke patients, pdBSI decreased in low frequencies and increased in high frequencies (*p* = 0.036) while DAR and DBATR were significantly associated with stroke severity (*p* = 0.0021 for DAR, *p* = 0.01 for DBATR). A classification tree model distinguished moderate/severe strokes from minor strokes and controls with 76% accuracy, 63% sensitivity, and 86% specificity. These findings suggest the potential of short EEG recordings for rapid stroke assessment [78].

The in-hospital phase of the ELECTRA-STROKE study (EEG-Controlled Triage in the Ambulance for AIS) used an eight-electrode dry-cap EEG system (Waveguard Touch, Eemagine, Berlin, Germany) to acquire raw EEG data in emergency settings. Recordings were later processed offline in Matlab to compute qEEG metrics. EEG data were collected from 100 ED patients with suspected stroke, of whom 9 (14%) had confirmed anterior LVO strokes (LVO-a). Among the qEEG features analyzed, the theta–alpha ratio (TAR) achieved the highest diagnostic performance (AUC: 0.83, sensitivity: 75%, specificity: 81%, NPV 97%), followed by Relative Alpha Power (AUC: 0.80). The combination of Relative Theta Power with the Weighted Phase Lag Index further enhanced performance, achieving 100% sensitivity and 84% specificity, supporting EEG as a promising tool for LVO-a stroke detection [75].

The prehospital phase of the ELECTRA-STROKE study evaluated real-time EEG processing in ambulances using the Waveguard Touch dry-cap system. Raw EEG data were recorded via a portable setup and processed online through NeuroCenter EEG software to compute qEEG metrics, including TAR and pdBSI. Among 212 analyzed patients, TAR achieved an AUC of 0.80 for LVO-a stroke detection (50% sensitivity, 83% specificity, 9% PPV, and 98% NPV), with pdBSI in the delta band having the highest accuracy (AUC 0.91, 80% sensitivity, 93% specificity, 24% PPV, and 95% NPV). These findings reinforce the potential of POC-EEG as an effective tool for early LVO stroke detection, enabling timely intervention even before hospital arrival [79].

#### 3.3.2. Evaluating Feasibility of POC-EEG Systems in Stroke Assessment

Here is a summary of six studies on the feasibility of POC-EEG systems used for stroke evaluation.

The BrainLab system, an eight-channel EEG system using Ag/AgCl electrodes placed according to the International 10–20 system, was employed for continuous monitoring in 21 acute hemispheric stroke patients (mean age 62–69 years). Feasibility outcomes demonstrated successful application within 24 h of admission, with recordings lasting 12–24 h and no significant technical issues [72].

The BrainScope system, a portable EEG device with five frontal electrodes, was tested for feasibility in 48 stroke patients (mean age 64.5 years) and 135 stroke mimics. Feasibility outcomes showed successful acquisition of artifact-free data within 10 min in 85% of recordings. Its portability and fast setup highlighted its practicality for emergency settings, making it suitable for acute settings [77].

The feasibility of the Muse device for EEG recordings was demonstrated in a study involving 25 participants. Minimal preparation was required using modified headbands and clip electrodes, achieving high connectivity and data quality [78].

In the other study with 58 participants, recordings were performed within 48 h post-stroke, also requiring minimal preparation. Despite challenges with artifacts and incomplete data points, the device consistently captured high-quality EEG signals, enabling the calculation of qEEG measures and supporting its usability in clinical settings [74].

The Waveguard dry electrode caps demonstrated feasibility and diagnostic utility in both in-hospital and prehospital settings. In-hospital studies reported median setup time of 2 min and recording duration of 3 min. However, 35% of recordings were excluded due to poor data quality, often caused by long hair or operator inexperience [75].

Prehospital studies highlighted rapid deployment by paramedics, with improvements in data quality over time, though challenges remained in long-hair patients and data exclusions (~32%). These findings support its potential for stroke triage [79].

### 3.4. POC-EEG Systems in Delirium Detection

#### 3.4.1. Evaluating the Diagnostic Accuracy and Clinical Implications of POC-EEG Systems for Delirium Identification

We identified seven studies on the diagnostic potential and clinical implications of POC-EEG devices in delirium assessment. Among them, two assessed the Ceribell rr-EEG device, two focused on the CMS2100 portable EEG device (CONTEC), one examined a simplified single-channel EEG setup, one analyzed a handheld bispectral EEG, and one evaluated the MobiMini POC-EEG system.

A multicenter study investigated postoperative delirium in 159 surgical patients using a simplified single-channel POC-EEG setup. EEG recordings were performed preoperatively and on the first three postoperative days alongside cognitive assessments. Delirium assessment was determined using a composite clinical reference standard, including video-recorded cognitive assessments. The device recorded raw EEG data, one minute of which was then processed in Matlab using spectral analysis to calculate relative power metrics. Relative Delta Power (RDP, 1–4 Hz) detected delirium with AUROC of 0.75, with improved performance (AUROC 0.78) when incorporating low-frequency power metrics (1–6 Hz). Additionally, RDP significantly correlated with delirium likelihood (*p* < 0.001), severity (*p* < 0.001), attentional deficits (*p* < 0.001), and level of consciousness (*p* < 0.001) [126].

A pilot study assessed a handheld bispectral EEG (BSEEG) device for delirium detection in the ED. The device recorded raw EEG data, which were post-processed to calculate a low-to-high frequency power ratio, forming the BSEEG index. Delirium assessment was determined using a composite clinical reference standard based on clinical impression and clinical delirium scales. Among 48 patients, the BSEEG index, at a cutoff of 1.45, demonstrated high accuracy in distinguishing delirious from non-delirious patients, with an AUROC of 0.91, a sensitivity of 88.9%, and a specificity of 92.3% compared to clinical diagnosis and cognitive assessment results. Study limitations included small sample size and limited generalizability to other settings [89].

Another study evaluated a handheld, two-channel POC-EEG device (CMS2100, Contec) for delirium detection in hospitalized patients. Delirium diagnosis was based on clinical delirium scales and expert psychiatric review. The device recorded 10-min EEG sessions twice a day using forehead electrodes. The power spectral density ratio algorithm was used to calculate the BSEEG index for delirium classification. In the training dataset (45 patients, 184 recordings), the BSEEG index at a cutoff of 1.44 identified delirium with 87.5% accuracy, 80% sensitivity, and 87.7% specificity (AUROC = 0.70). Validation on 24 additional cases yielded consistent results, with 83.3% accuracy, sensitivity, and specificity (AUROC = 0.81) [90].

A large prospective study employed a handheld, two-channel POC-EEG device (CMS2100, Contec) to evaluate delirium and outcomes in 274 of 428 hospitalized patients. Delirium diagnosis was based on clinical delirium scales and expert psychiatric review. Raw EEG data were analyzed post hoc using spectral density analysis to generate a BSEEG score, which demonstrated a strong association with clinical delirium and adverse patient outcomes. Specifically, higher BSEEG scores correlated with longer hospital stays (*p* = 0.0014), discharge to non-home settings (*p* = 0.009), and increased mortality (HR = 1.35, *p* = 0.025). Additionally, elevated BSEEG scores predicted mortality better than clinical delirium status, identifying high-risk patients undetected by conventional methods [88].

Delirium superimposed on dementia (DSD) poses unique diagnostic challenges due to overlapping symptoms and psychotropic medication effects. A study on 30 dementia patients, 14 with DSD, evaluated the MobiMini POC-EEG system using bipolar recordings from the right prefrontal and temporal areas. Delirium diagnosis was based on clinical scales and expert review of video-recorded behavioral assessments. Raw EEG data were processed post hoc using BrainVision Analyzer to analyze power spectral density. The Delta/Theta2 (6–8 Hz) power ratios achieved an AUC of 0.80 (*p* < 0.001) to distinguish DSD from dementia alone, with a sensitivity of 90% and a specificity of 40%. However, all patients received benzodiazepines, and half were on clozapine, potentially affecting results and warranting further validation [127].

A prospective pilot study included 13 critically ill ICU patients (≥50 years, mechanically ventilated >12 h). Delirium diagnosis was based on researcher-conducted clinical assessments. Ceribell rr-EEG recorded 2 h sessions for up to 4 days, with data preprocessed in Matlab. Gamma/delta and gamma/theta power ratios were analyzed using machine learning algorithms. Stepwise Linear Discriminant Analysis (SWLDA) achieved the highest classification accuracy (97–98%) for differentiating delirium-positive and -negative participants. Support Vector Machines (SVMs) demonstrated lower accuracies (74–82%), while random forest ~70%. Gamma power ratios consistently differentiated delirium-positive from -negative cases, supporting machine learning-assisted EEG as a potential delirium biomarker [86].

A pilot study tested Ceribell rr-EEG system combined with a supervised deep learning Vision Transformer (ViT) model to detect delirium in 13 critically ill ICU patients (≥50 years old, mechanically ventilated ≥12 h). Delirium diagnosis was based on clinical scale assessments. The ViT model was applied to the preprocessed EEG data, classifying patients as delirium-positive or -negative. EEG waveforms were converted into image-like data slices of varying sizes (e.g., 5 s slices) for classification. The ViT model achieved 97.58 testing accuracy with optimal data slices. Importantly, longer wave cycles, reflecting delta activity, were more predictive of delirium. This approach outperformed traditional frequency–domain EEG analyses, reinforcing the role of low-frequency signals (e.g., delta waves) as key predictors. These findings highlight the potential of ViT and rr-EEG for high-accuracy delirium detection [128].

#### 3.4.2. Evaluating the Feasibility of POC-EEG Systems for Delirium Identification

Here is a summary of five studies evaluating the feasibility of POC-EEG systems for assessing delirium.

The handheld, two-channel POC-EEG device (CMS2100, Contec) demonstrates high operational feasibility due to its ease of use and minimal setup requirements. Electrodes are placed on the forehead and earlobes, enabling a 10 min recording session that allows efficient data collection without disrupting clinical workflow. The device supports sustainability by enabling twice-daily recordings as part of routine care. Standardized signal processing ensures consistent BSEEG score calculation, enhancing reliability for clinical applications [88].

A handheld BSEEG device was successfully used in an ED, acquiring EEG data from 48 patients. The device featured a simplified two-channel setup, and a trained research fellow provided initial guidance for its application [89].

In another study, the operational feasibility of the handheld, two-channel POC-EEG device (CMS2100, Contec) was further validated. The portable device required only five electrodes for quick setup, with signal quality comparable to traditional 20-lead EEG systems, as confirmed by an EEG expert. The protocol proved sustainable, with recordings conducted twice daily for up to seven days, with extensions if changes in the patient’s mental status were observed. The study also highlighted seamless integration into clinical workflows, including successful data transfer to a secure server for processing and analysis [90].

The operational feasibility of a single-channel EEG for delirium evaluation has been demonstrated through several factors: the successful integration of a 1 min automated EEG analysis into postoperative care workflows, achieving 92.3% of patient participation and 92.9% of expected recordings successfully obtained despite technical challenges. Data collection was streamlined through a standardized protocol for EEG recordings and cognitive assessments. Additionally, the system required minimal training for researchers, with only 60 min of theoretical and practical instruction, making it easy to implement in clinical settings [126].

The operational feasibility of MobiMini was demonstrated through the successful acquisition of 49 EEG recordings in a psychogeriatric unit, despite patients experiencing severe agitation and psychotic symptoms. Additionally, low discomfort scores during assessments highlighted the protocol’s acceptability in this vulnerable population [127].

## 4. Discussion

This review provides a comprehensive synthesis of the current literature on the use of POC-EEG in the management of emergency neurological conditions in adults, focusing primarily on its diagnostic accuracy, operational feasibility and clinical implications in conditions such as NCSE, TBI, stroke and delirium.

### 4.1. Diagnostic Accuracy, Feasibility, and Clinical Implications of POC-EEG Systems in the Assessment of NCSE

POC-EEG has been extensively studied for rapid neurophysiological assessment in suspected NCSE [9,33,35,37,94,95,96,99]. Its feasibility, portability, and rapid accessibility make it highly suitable for time-sensitive scenarios, particularly in resource-limited settings where conv-EEG is not immediately available [6,7,8,33,34,36,107]. It has also been widely integrated into critical care settings [8,9,36,94,95,96,99] and has proven useful in COVID-19 isolation units, minimizing staff exposure [27,36,101]. POC-EEG systems are designed for rapid deployment with simplified setup procedures that enable use by neurology residents, emergency physicians, and intensivists [7,9,37,97,99]. Setup times typically range from 5 to 10 min, significantly faster than conv-EEG, which can require several hours due to technician and equipment availability [27,29,32,94,98,99]. In emergency settings, POC-EEG has significantly reduced door-to-EEG initiation time, allowing recordings to begin within minutes, whereas conv-EEG is often delayed by hours or even days [5,6,8,9,37,94,105]. Faster interpretation times have also been reported, with diagnoses available minutes to hours earlier than with conv-EEG [7,27,94]. POC-EEG systems are often conducted after hours, with rates ranging from 55% to 69% [6,8]. Their availability reduces reliance on EEG technicians and improves accessibility in centers where conv-EEG is not available 24/7 [6,8,27,32,99]. The implementation of POC-EEG has been associated with a higher volume of EEG studies performed, with some studies reporting an increase of up to three times compared to pre-implementation levels [5,6,8]. Additionally, it has led to an increased diagnostic yield compared to standard care [5,6,8,99]. Studies indicate that after-hours recordings frequently detected seizures, with up to 90% of seizure-detecting POC-EEGs performed after hours, reinforcing the need for immediate EEG accessibility in time-sensitive cases [6,8]. The integration of POC-EEG into telemedicine platforms has further enhanced accessibility, enabling real-time remote expert review [8]. Patient tolerability is high, with only minor, self-limiting adverse events, such as mild scalp irritation, supporting its safety [6]. Signal quality is generally comparable to conv-EEG [100,103], with minimal artifacts, though electrical interference remains a limitation, particularly in longer recordings [97,98,107] as well as in certain populations or settings [29].

The diagnostic performance of POC-EEG in detecting ESE/SENC varies across studies due to differences in electrode configurations, interpreter expertise, and technology-assisted analysis. Additionally, study designs, patient populations, recording durations, and reference standards contribute to the heterogeneity of findings [33,34,35,95,96,104,106,107]. Detection rates for ictal or ictogenic patterns beyond SENC, such as seizures, ESz, and HEPs, vary significantly and are influenced by the classification methodology used in analysis [7,31,96,97,98,99,107]. Spatial coverage seems to influence POC-EEG accuracy. A study found that forehead-only electrode setups had limited sensitivity (50%) for SE detection due to missed posterior activity, despite achieving 100% specificity [95]. In contrast, devices with broader spatial coverage have shown higher sensitivity (70.6%) with strong specificity (97%) [96]. Another study using a longitudinal external montage found that seizures in critically ill patients were not confined to the midline or parasagittal region, indicating that this montage did not compromise diagnostic accuracy [103].

Real-time interpretation methods, such as EEG sonification, have enhanced interpretability, especially for non-EEG-trained physicians. In a small group, POC-EEG with sonification demonstrated high sensitivity for SE detection, correctly identifying 1/1 SE case and ruling out 9/10 non-SE cases, with one false positive later corrected by conv-EEG [27]. One study found that emergency physicians using auditory EEG sonification detected seizures with 100% sensitivity and 92% specificity [7]. Another study reported that sonified POC-EEG and its visual review increased seizure detection sensitivity from 77.8% (clinical suspicion alone) to 100% for neurology-trained physicians [9]. Similarly, among ICU neurointensivists without epilepsy training, sonified EEG improved seizure detection from 0% to 100%, though in a small sample size [37]. Non-specialist operators, such as medical students and nurses, achieved acceptable accuracy with brief training, particularly when using EEG sonification. However, experienced neurophysiologists demonstrated the highest diagnostic accuracy [31]. Despite its effectiveness in detecting ictal patterns, EEG sonification struggled to differentiate seizure-like rhythmic or PDs such as GPDs, LPDs, and triphasic waves [31]. Additionally, it had limited coverage and difficulty distinguishing seizure-like patterns such as artifacts and rhythmic movements, potentially leading to misinterpretations [37].

AI-assisted detection tools, which generate alerts when SzB exceeds a predefined threshold, further influence performance. The algorithm has shown reliable performance at the extremes of the SzB spectrum—namely for SE and normal/diffusely slow activity—but is less effective for detecting milder abnormalities [33]. One study showed that the latest version of the AI algorithm (version 6.0) exhibited 86% sensitivity for detecting ESE/pESE at a low SzB threshold (>10%), making it a reliable screening tool for ruling out critical cases (NPV of 99%). When the threshold was set at >90%, specificity increased to 99%, with a PPV of 78%, making it useful for confirming such conditions and prompting immediate intervention [107]. Another study reported a 6.8% false-positive detection for SE alert at > 90% SzB, with most cases (87.5%) later reclassified as HEPs upon expert review [33]. Additionally, at a ≥90% SzB threshold, the algorithm missed one SE case (false negative) and generated 17 false positives out of 665 POC-EEG recordings, with only one misclassified as non-epileptiform activity [35]. AI-based interpretation showed limited sensitivity for isolated discrete ESz and HEPs, detecting only 54% of such events at lower SzB thresholds [107]. However, updated versions of the tool have demonstrated improved diagnostic performance, paving the way for more accurate seizure detection systems [35].

Several studies have demonstrated that POC-EEG increases diagnostic yield, reinforcing its value in acute and critical care settings [5,6,8,99]. Its implementation has been linked to higher seizure detection rates, capturing more seizures and seizure-like activity compared to standard approaches [5]. One study reported that POC-EEG identified the same or additional pathological EEG patterns in 94.5% of cases, including IEDs, focal slowing, and generalized slowing. Additionally, in 9.3% of patients without immediate access to conv-EEG, POC-EEG detected clinically significant findings such as IEDs and SE, influencing clinical management [99]. In ICU settings, POC-EEG identified epileptiform discharges and seizures in 89% of cases, with a 98% agreement with conv-EEG for seizure detection. It also increased the detection rate of epileptiform discharges from 15% (conv-EEG) to 34%, demonstrating its ability to capture evolving patterns that might be missed with delayed conv-EEG [98].

POC-EEG recording durations vary widely, ranging from short screening sessions (10–20 min) [94,95] to extended recordings up to 12 h [32]. Short-duration POC-EEG [94,95] is primarily used for emergency triage, while prolonged recordings provide a bridge to conv-EEG [32] or are applied to ICU patients who require extended monitoring [6,8,32,33,96,97,98,104]. One study showed that 94% of ESz and HEPs and 100% of definite seizures were detected within the first two hours of monitoring, with 70% identified within the first 10 min, highlighting the ability of short-duration POC-EEG to rapidly capture critical EEG abnormalities and supporting its use in triage [107]. The role of POC-EEG in triaging and risk stratification was further reinforced by a study showing that 1 h POC-EEG provided seizure risk prediction comparable to conv-EEG using the 2HELPS2B scoring system, effectively distinguishing high-risk patients requiring prolonged monitoring from lower-risk cases and optimizing resource allocation [102]. Additionally, seizure-relevant EEG patterns detected in the first hour of POC-EEG were consistent with findings on follow-up conv-EEG, underscoring the reliability of early POC-EEG assessment in guiding clinical decisions [102].

Patient-specific factors also influence POC-EEG performance. Most studies focus on suspected NCSE from various causes, while few have examined PCA patients. In this population, AI-assisted detection tools failed to detect any ictal abnormalities, highlighting reduced sensitivity in this subgroup [104]. Additionally, expert-interpreted POC-EEG and conv-EEG showed only fair agreement, emphasizing the need for further research [29]. Conversely, POC-EEG has been successfully integrated into acute stroke workflows, aiding in ESE detection and stroke mimic evaluation without delaying management [106].

POC-EEG has significantly impacted clinical decision-making, influencing diagnosis, treatment, patient management, hospital LOS, and resource utilization. It significantly enhances clinical confidence in diagnosing and treating suspected NCSE, reducing reliance on clinical judgment alone [9]. In ICU settings, 50% of POC-EEG patients received ASM, compared to 35% in the conv-EEG group [98]. Studies have shown that POC-EEG and its integrated tools influence clinical decisions across different settings. In the ICU, EEG sonification altered seizure suspicion in 40.2% of cases and impacted treatment decisions in 20.1% [9], while in the ED, it led to management changes in 53% of patients [7]. In the ED, the combination of POC-EEG and AI-assisted tools resulted in ASM adjustments for 59.2% of cases, with 50% receiving necessary initiation and 26.7% avoiding unnecessary escalation [6]. Similarly, in the ICU, sonification-driven treatment modifications occurred in 40% of cases, reducing unnecessary ASM use by 27% [37]. In one case, POC-EEG findings prevented unnecessary intubation and aggressive therapy [37]; while in two cases, they led to a transition from aggressive treatment to palliative care [27]. While POC-EEG detects additional pathological activity compared to conv-EEG, optimizing therapeutic interventions [98], its effect on outcomes varies. One study showed a trend toward improved functional outcomes [105] but no significant differences in survival curves between POC-EEG and conv-EEG over 72 h [102] or in mortality outcomes [105].

POC-EEG has demonstrated economic advantages, particularly by reducing hospital transfers, shortening LOS, and optimizing resource allocation [5,6,7,8,105]. More efficient ASM management prevents unnecessary or empirical medication use [6]. POC-EEG has been associated with a reduction in ICU and hospital LOS. One study reported a significant decrease in ICU LOS from 8.0 to 4.5 days in critically ill patients with seizure concerns [105], while another showed a trend toward shorter hospital LOS post-implementation [5]. Additionally, EEG sonification expedited patient disposition in 21% of cases [7]. Door-to-EEG time was positively correlated with longer ED and hospital LOS [6]. The financial impact of shorter hospital stays has been estimated at annual cost savings of ~USD 737,818, with an additional USD 145,580 from DRG reimbursements [5]. In community hospital settings, POC-EEG significantly reduced hospital transfers, leading to net financial savings. One study found transfers decreased from 2 to 1.1 per month after POC-EEG implementation, saving USD 13,936 per patient, effectively covering fixed device costs [36]. Another study, which combined POC-EEG with a hub-and-spoke tele-EEG system, reduced transfers from 44.6% to 5%, preventing unnecessary transfers in 94% of cases and generating cost savings exceeding USD 39,000 [8].

### 4.2. Diagnostic Accuracy, Feasibility, and Clinical Implications of POC-EEG Systems in the Assessment of TBIs

The qEEG indices, derived from POC-EEG recordings and machine learning analysis, have shown strong performance in identifying traumatic structural brain injuries (>1 mL hematomas) [28,49,113] and functional impairments (concussions) [109,117,120,121]. Some classifiers have shown high accuracy in identifying CT+ cases, with an AUC of 0.82, indicating strong discriminative ability. Sensitivity consistently exceeds 90% with an NPV of 96%, which ensures high confidence in ruling out structural injuries. However, specificity remains moderate (51.6%), leading to a substantial number of CT+ cases being misclassified [49]. Additionally, qEEG-derived composite indices have identified intracranial hematomas in mTBI patients, regardless of lesion location or electrode distance, with sensitivity ranging from 95% to 100% and specificity between 44% and 66%, offering high confidence in ruling them out (NPV 100%) [28,113]. The qEEG classifiers showed higher specificity than the clinical decision rules NOC and NEXUS II for detecting acute mTBI while maintaining similar sensitivity, whereas CCHR had greater specificity (86.5%) but significantly lower sensitivity [57,114]. POC-EEG-based classifiers have demonstrated a 26–30% reduction in CT referrals compared to standard clinical practice, optimizing imaging use and reducing radiation exposure [47,118]. One study reported a 33% reduction in false-positive CT scans compared to traditional clinical evaluation [47]. Even in substance-intoxicated patients, POC-EEG reduced false-positive CT referrals by 29.3%, improving triage efficiency without compromising sensitivity [119].

The qEEG-based classifiers also aid in early concussion detection [50,117,120,121] and severity grading [48,56,109], with sensitivities up to 86% and specificities up to 70% in differentiating concussed individuals from controls, though most studies focus on young adults [117,121]. Additionally, POC-EEG tracks electrophysiological recovery, even after clinical symptom resolution [55,56,110,111,117,120,121], and predicts RTP timelines in athletes [56,117,121,122]. Links between qEEG markers and DTI metrics suggest a role in assessing white matter integrity, although small sample sizes, lack of control groups, and limited longitudinal follow-up constrain these findings [54].

The non-invasive, portable design and setup time of under five minutes support POC-EEG integration into EDs, prehospital care, and military settings, with successful use by non-specialized personnel [28,47,55,57,108,109,110,111,113,114,118,122,123].

The qEEG-based indices derived from post-acquisition processing have also been used to differentiate brain-dead TBI patients from survivors and correlate with serum biomarkers, offering potential for neurological deterioration monitoring [30,125]. However, further studies are needed to evaluate the clinical impact of POC-EEG in TBI, particularly on hospital stay duration and healthcare costs.

### 4.3. Diagnostic Accuracy, Feasibility, and Clinical Implications of POC-EEG Systems in the Assessment of Strokes

POC-EEG systems have demonstrated moderate-to-high diagnostic accuracy for LVO-a stroke detection in emergency prehospital settings with TAR achieving an AUC of 0.80 and pdBSI an AUC of 0.91. High NPV (98–99%) establishes POC-EEG as a strong rule-out tool for LVO-a stroke, emphasizing its potential for stroke triage [79]. Rapid setup (≤3 min) and portability enable use by non-specialized personnel, though data quality issues (32–35% exclusions) remain a limitation [75,79]. However, the limited number of studies on POC-EEG real-time performance in stroke care keeps this field open for further research [79].

Post-acquisition processing and machine learning classifiers enhance POC-EEG diagnostic utility, though their effectiveness depends on the specific processing methods used [72,74,75,78]. EEG markers like BSI, rsBSI, and pdBSI distinguish stroke patients from controls, correlating with severity and functional recovery outcomes [72,74]. Additionally, EEG indices reflecting signal slowing, such as DAR and DBATR, further validate their diagnostic and prognostic values in both acute and post-acute stroke phases [78].

### 4.4. Diagnostic Accuracy, Feasibility, and Clinical Implications of POC-EEG Systems in the Assessment of Delirium

POC-EEG systems show potential for delirium detection, with diagnostic performance largely relying on post-acquisition processing [86,88,89,90,126,127,128]. Studies using POC-EEG combined with spectral analysis have identified qEEG markers, such as low-frequency power metrics (e.g., delta and theta waves) and the low-to-high frequency ratio (BSEEG index), as being associated with delirium [88,89,90,126]. The BSEEG index demonstrated high diagnostic accuracy with an AUROC of 0.91, sensitivity of 88.9%, and specificity of 92.3% [89]. Additionally, BSEEG scores correlate with prolonged hospital stays, non-home discharges, and increased mortality, suggesting both clinical and prognostic relevance [88]. However, psychotropic medications may affect accuracy, as observed in studies on patients with dementia [127]. Machine learning models, particularly ViT-based deep learning, achieved 97.58% accuracy in delirium detection, outperforming traditional EEG frequency-domain analyses and supporting gamma and delta power ratios as key delirium biomarkers [128]. These findings emphasize the importance of combining POC-EEG data acquisition with sophisticated post-acquisition algorithms to enhance diagnostic precision and reliability [86,128].

Feasibility studies highlight rapid setup, minimal training requirements, and effective bedside use in EDs, ICUs, and psychogeriatric units, even in patients with severe agitation or psychosis [89,90,127]. The portability of POC-EEG facilitates integration into clinical care, allowing frequent recordings and both continuous and intermittent monitoring [90].

Despite promising results, small sample sizes, medication effects, and reliance on post hoc analyses warrant further validation in multicenter trials [27,86,88,89,90,127,128].

### 4.5. Limitations

This review has limitations typical to narrative reviews. The heterogeneity of study designs, patient populations, POC-EEG systems, and clinical settings limits the ability to quantify diagnostic performance and generalize findings across all contexts. Variability in device specifications, electrode configurations, and post-acquisition processing methods further contributes to inconsistencies. To address this limitation, we conducted a descriptive analysis to identify key trends, common findings, and areas of variability.

The relatively limited number of high-quality studies in certain subpopulations, such as patients with post-anoxic brain injury, restricts generalizability and highlights the need for further research. The lack of formal quality assessment prevents a systematic evaluation of bias. Additionally, the exclusion of non-English studies and potential publication bias may have influenced the overall portrayal of POC-EEG diagnostic accuracy.

## 5. Conclusions

POC-EEG is a valuable screening tool in acute neurological care, offering high predictive value, clinical utility, speed, simplicity, broad availability, and cost-effectiveness in specific applications. It enables rapid diagnosis and risk stratification in time-sensitive conditions, improving patient outcomes and optimizing resource allocation. Its accessibility, ease of use by non-specialists, and telemedicine integration make it particularly useful in resource-limited settings.

However, challenges remain, including variability in diagnostic accuracy and reliability in certain populations, the lack of real-time technology-assisted optimization in stroke and delirium, and technical limitations associated with reduced electrode arrays. Addressing these issues requires hardware improvements, real-time AI integration, and expanded tele-EEG networks to enhance reliability and support broader clinical adoption.

Beyond its current applications, POC-EEG has significant potential for prehospital triage in time-sensitive neurological conditions such as NCSE and stroke, facilitating earlier intervention. In ICU settings, continuous monitoring could improve decision-making, while in severe acquired brain injuries, it may aid in prognostication, extending its role beyond acute care.

## Figures and Tables

**Table 1:** Overview of published studies on POC-EEG use in NCSE.

	Study Design	Feasibility	Diagnostic Performance	Clinical Implications and Cost-Effectiveness
1.	*First author*: Brenner [94], 2015, USA *Sample size*: 12 adult patients (median age: 51.5) *Conditions*: AMS with seizure history or witnessed seizure *Setting*: ED *EEG System*: Portable Brainmaster EEG device *Comparison*: conv-EEG (reference standard) *EEG Interpretation*: neurophysiologist (POC-EEG); neurologist (conv-EEG)	POC-EEG: *Time from consent to EEG start*: median 10 min *Time from EEG ordering to interpretation*: median 38 min *Recording duration*: standard *Connectivity*: no wireless, wired internet port necessary for remote access	Agreement between POC-EEG and conv-EEG: NCSE determination: 100% agreement NCSE POC-EEG diagnostic rate: 1 out of 12 with NCS, 0 NCSE	POC-EEG device cost: ~USD 2500 conv-EEG cost: ~USD 50,000
2.	*First author*: Muraja-Murro [95], 2015, Finland *Sample size*: 100 patients (18–90 aged) *Conditions*: unexplained AMS from various etiologies *Setting*: ED *EEG System*: Grass Technologies *Comparison*: fm-EEG (reference standard) *EEG Interpretation*: Three expert neurophysiologists, blinded to EEG type	POC-EEG: *Recording duration:* standard (conv-EEG: 20 min simultaneous recordings) *Recording quality*: 2% of recordings unsatisfactory due to artifacts	POC-EEG performance: SE detection: Se 50%, Sp 100%, accuracy 98%; PPV 100%, NPV 98%, FP 0; no; FN 2/4 (SE cases missed due to posterior brain activity beyond the recording area)	
3.	*First author*: Rittenberger [29], 2019, USA *Sample size*: 95 patients (mean age: 59) *Condition*: PCA *Setting*: Tertiary care cardiac arrest hospital *EEG system*: Cadwell 6-electrode POC-EEG *EEG Interpretation*: Epileptologist and neurointensivist *Comparison*: First 30 min of c-conv-EEG, performed after POC-EEG *EEG interpretation*: Epileptologist and neurointensivist	POC-EEG: *EEG setup*: providers without specialized EEG training *Mean door-to-EEG time*: 96 min *Mean duration*: 354 min *Uninterpretable*: 40% due to electrical interference (vs. conv-EEG: 13%)	Agreement between POC-EEG and c-conv-EEG: Cohen’s kappa = 0.27 (fair agreement) EEG findings: *Most common POC-EEG patterns*: - Continuous: 21% - Generalized suppression: 14% - Burst suppression: 12% - Burst suppression with identical bursts: 10% - Seizures: 2% (2/95) *Most common cEEG patterns*: - Generalized suppression: 31% - Burst suppression with identical bursts: 27% - Continuous: 18% - Seizures: 2%	Survival to hospital discharge: - POC-EEG findings (*p* = 0.19) - conv-EEG findings (*p* = 0.11) Proximate cause of death: - POC-EEG findings (*p* = 0.14) - conv-EEG findings (*p* = 0.8)
4.	*First author*: Egawa [96], 2020, Japan *Sample size*: 50 patients (median age: 72) *Conditions*: AMS from various etiologies (subarachnoid hemorrhage, cerebral hemorrhage, post-cardiac arrest (PCA) syndrome, SE, TBI) *Setting*: neuro-ICU *EEG system*: AE-120A EEG headset *Comparison*: fm-cEEG (immediately subsequent) *EEG Interpretation*: One neurointensivist and one board-certified neurophysiologist	POC-EEG: *Recording duration*: cEEG monitoring *Connectivity*: bluetooth connection for simultaneous EEG data transmission *Time-to-EEG*: median 57 min (reduction of 303 min) *Monitoring duration*: median 134.5 Feasibility for critical care environments	POC-EEG findings: - Detection of abnormal pattern (PDs, RDA, SW, CS): Se 97.4%, Sp 90.9% - Detection of NCSE: Se 70.6%, Sp 97.0% - Detection of PD: Se 82.4%, Sp 97.0% - Detection of CS: Se 90%, Sp 97%	
5.	*First author*: Caricato [97], 2020, Italy *Sample size*: 40 patients - Study group: 20 neuro-ICU - Control group: 20 general ICU *Conditions*: Subarachnoid hemorrhage, cerebral hemorrhage, head injury *EEG system*: CerebAir headset (study group) *Comparison*: 8-electrode rm-EEG (control group) (continuous) *EEG Interpretation:* Expert neurologist	EEG Application: - POC-EEG: neuro-ICU physician - rm-EEG: EEG technician Electrode application time: - POC-EEG: 6.2 ± 1.1 min - rm-EEG: 10.4 ± 2.3 min (*p* < 0.0001) Monitoring duration: - POC-EEG: 57 ± 12 h - rm-EEG: 75 ± 15 h Artifact corrections needed: - POC-EEG: 35 - rm-EEG: 11 (*p* < 0.01) Skin reactions: - POC-EEG: Mild pressure lesions in 17; monitoring discontinued in 4 cases after ~52 h due to risk of worsening skin damage	POC-EEG findings: EEG abnormalities classified as - EA: Generalized/focal seizures, SE, GPDs, LPDs - Non-EA: Focal/generalized slow waves, sharp waves, EEG asymmetries EEG abnormalities were detected in both groups at similar rates (*p* > 0.05): - Study group: 35% EA, 35% non-EA - Control group: 25% EA, 45% non-EA	EEG-related ASM initiation: - Study group: 50% - Control group: 35%
6.	*First author*: Meyer [98], 2021, Germany *Patients*: 52 patients (mean age: 63 years) *Conditions*: AMS due to SE, ischemic stroke, intracranial bleeding, meningitis, encephalitis, metabolic encephalopathies *Setting*: neuro-ICU *EEG system*: CerebAir monitoring *Comparison*: Routine conv-EEG (delayed) *EEG Interpretation*: Resident physician, supervised by a board-certified senior physician	POC-EEG: *Duration*: long-term monitoring *Technical Issues*: electrode detachment and impedance problems in few cases (some planned cEEG data could not be fully evaluated due to electrode instability)	Diagnostic Performance: *Detection of epileptiform discharges*: - POC-EEG: 34%; rEEG: 15%; agreement: 68% *Detection of seizures*: - POC-EEG: 4%; rEEG: 2%; agreement: 98% *Detection of ICU-relevant patterns (epileptiform discharges or seizure)*: - POC-EEG: 34%; rEEG: 11%; POC-EEG detected 89% of the same or additional ICU-relevant EEG patterns compared to conv-EEG *Detection of Background activity*: - Agreement: 98%	
7.	*First author*: Welte [99], 2024, Germany *Sample size*: 100 patients *Setting*: Neurological ED *Conditions*: AMS or suspected seizures *EEG system*: CerebAir (minimum 10 min) *EEG Interpretation*: Neurology resident, supervised by an EEG expert *Comparison*: conv-rEEG (55 patients) performed immediately hours to days after POC-EEG *EEG Interpretation*: Specialized neurology residents, supervised by senior board-certified EEG experts	POC-EEG: *Setup time*: median 7 min *EEG setup*: non-EEG-trained medical students *Recording duration*: > 10 min *Data transmission*: bluetooth to a bedside laptop *After-hours availability* *Technical issues*: frequent impedance issues, especially in occipital electrodes Application challenges: long/thick hair, uncooperative patients (74 cases needed repositioning or extra gel application)	Agreement between swEEG and first rEEG results (55 patients): - General EEG findings (normal vs. pathological slowing vs. epileptiform activity): 87.3% - POC-EEG identified same or additional pathological patterns (IEDs, focal slowing, generalized slowing): 94.5%	Potential therapeutic intervention: - In 9.3% of patients without rEEG or with delayed rEEG, POC-EEG identified clinically significant findings (IEDs, SE) that could have led to therapeutic interventions
8.	*First author*: Hobbs [37], 2018, USA *Sample size*: 34 patients (mean age: 61) *Setting*: ICU *Condition*: AMS (GCS <12) due to mixed etiologies (metabolic encephalopathy, AIS, intracerebral hemorrhage, TBI, and autoimmune encephalitis) and requiring EEG monitoring *EEG system*: Ceribell rr-EEG system *EEG Interpretation*: Sonified EEG by neurointensivists without epilepsy training *Comparison*: conv-EEG performed after POC-EEG; reference standard *EEG Interpretation*: Two epileptologists reviewed the entire Ceribell EEG recording and correlated findings with conv-EEG reports	POC-EEG: *EEG application*: ICU physicians without EEG training *Setup Time*: 6 ± 3 min *Time-to-EEG*: median 23 min (vs. conv-EEG: median 145 min; *p* < 0.001) *Recording duration*: monitoring continued until conv-EEG was initiated (vs. conv-EEG: median 18 h) *Ease-of-use rating (scale 1–5)*: headband: 4.51 ± 0.85; device: 4.97 ± 0.17 *Connectivity*: wirelessly for remote review	Diagnostic Performance: *Seizure detection sensitivity*: - Clinical suspicion alone: 0% (0/2) - Ceribell sonification: 100% (2/2) *Seizure detection specificity*: - Clinical suspicion: 76% - Ceribell sonification: 85%	Sonification tool impact: - Treatment decision changed in 40% of cases - Prevented unnecessary treatment: 21–27% - Avoided intubation/ more aggressive therapy in 1 case
9.	*First author*: Parvizi [31], 2018, USA *Sample size*: 84 EEG samples selected from patients *Condition*: AMS *EEG system*: Ceribell (visual + sonification) *EEG Interpretation*: - Visual EEG: Neurologists (*n* = 12) and medical students (*n* = 29) - Audio EEG: 34 medical students and 30 nurses *Reference standard*: Full rr-EEG reviewed by three epileptologists	EEG sonification: *Short training required*: 4 min instructional video before testing	Diagnostic Performance: *Audio EEG Seizure Detection*: - Students: Se 98%; Sp 65% - Nurses: Se 95%; Sp 66% *Audio EEG seizure/seizure-like events detection*: - Students: Se 79%; Sp 85% - Nurses: Se 77%; Sp 82% *Visual EEG seizure detection*: - Neurologists: Se 88%; Sp 87% - Students: Se 76%; Sp 65% *Visual EEG seizure/seizure-like events*: - Neurologists: Se 88%; Sp 90% - Students: Se 62%; Sp 65%	
10.	*First author*: Yazbeck [32], 2019, USA *Sample size*: 10 patients (mean age: 59.7 years) *Setting*: neuro-ICU *Condition*: AMS at risk for NCSE *EEG system*: Ceribell rr-EEG system sound application; interpreted on-site by treating physicians using real-time sonification and visual review on the rr-EEG device *Comparison*: conv-EEG performed after POC-EEG in six patients	POC-EEG: *EEG setup time*: 5 min (vs. conv-EEG: 11.2 h, *p* < 0.001; vs. after-hours conv-EEG cases: median delay of 12.29 h) *EEG application*: ICU staff *Recording duration*: varying, with monitoring continuing for up to 12 h unless conv-EEG became available *Ease-of-Use Ratings (Scale 1–5, Lower = Easier)*: headband: 1.9 ± 1.0; device: 1.6 ± 0.8	Concordance with conv-EEG: - 5/5 cases agreement for ruling out seizure - 4/5 high-suspicion cases reassessed as low suspicion - 1 case inconclusive due to artifact	POC-EEG Impact on treatment decision: - POC-EEG prevented additional unnecessary ASM treatment in 40% (4/10) cases
11.	*First author*: Kamousi [100], 2019, USA *Sample size*: - 1 healthy subject for laboratory simultaneous recordings - 22 ICU patients *Settings*: Laboratory setting and neuro-ICU *Conditions*: AMS (ICU study); healthy subject component (controlled laboratory setting) *EEG system*: Ceribell rr-EEG system *Study design*: - Simultaneous EEG recordings (laboratory setting) using rr-EEG and two conv-EEG systems - Consecutive EEG recordings (ICU study), where rr-EEG was applied first, followed by conv-EEG	Laboratory Study (Healthy Subject): *Waveform correlation between rr-EEG and conv-EEG*: statistically equivalent ICU study: *Metrics comparison*: - No significant difference in Hjorth parameters (activity, mobility, complexity) between rr-EEG and conv-EEG - No significant difference in artifact-related measures (baseline wander, artifact spike count, kurtosis) - Significant reduction of 60 Hz noise with rr-EEG (*p* = 0.0005)		
12.	*First author*: Chen [101], 2020, USA *Sample size*: 5 patients *Setting*: ICU *Conditions*: AMS or suspected seizures or SE in critically ill adult patients with confirmed COVID-19 infection *EEG System*: Ceribell rr-EEG *Comparison*: conv-EEG performed in 2 patients for extended monitoring	rr-EEG was successfully deployed in all 5 ICU patients Setup was feasible in an isolation setting, reducing healthcare personnel exposure risk		
13.	*First author*: LaMonte [27], 2021, USA *Sample size*: - Study group: 10 patients - Control group: 40 patients *Conditions*: suspected SE, including cases of PCA, AMS, Central Nervous System (CNS) lesions and epilepsy *EEG system*: - Study group: Ceribell rr-EEG system; interpretation: on-call neurologist using sonification and visual review, duration - Control group: conv-EEG (routine or continuous)	POC-EEG: *EEG application*: nurses, respiratory therapists, or EEG technologists *Recordings duration*: standard (up to 2 h in some cases) *Connectivity*: Wi-Fi portal with sonification analysis to real-time analysis *Setup time*: <10 min *After-hours technologist call-ins*: 0 (vs. conv-EEG: 15/40 cases, *p* = 0.02) *Deployment*: successful in all hospital areas, including COVID-19 isolation rooms *Technical issues*: minor impedance issues, resolved with adjustments *Storage and cleaning*: Easy to disinfect and reuse in isolation settings	POC-EEG Diagnostic Performance: *Time to Diagnosis*: 23.8 min (vs. conv-EEG: 126.5 min, *p* = 0.0000006) *SE diagnosis:* - 1/10 cases: confirmed SE diagnosis - 9/10 cases: ruled out SE diagnosis *Concordance with conv-EEG*: - 6 of 7 cases (85.7%) had identical SE vs. SE diagnosis - 1 case: false-positive SE diagnosis led to immediate treatment; the follow up conv-EEG (30 min later) ruled out seizure activity	POC-EEG implication: Oriented Medication Use: prevented empirical treatment in 1/10 case with SE at POC-EEG Palliative Care Decisions: 2/10 cases resulted in transition from aggressive treatment to palliative care based on POC-EEG findings
14.	*First author*: Vespa [9], 2020, USA *Sample size*: 181 patients (mean age: 58.6) *Setting*: ICUs from five academic hospitals *Condition*: AMS suspected of NCS *EEG system*: Ceribell rr-EEG system (30 s sonification per hemisphere + 60 s visual EEG review; real-time interpretation: treating physician; remote neurologist review) *Comparison*: conv-EEG performed immediately after POC-EEG; reference standard	POC-EEG: *EEG application*: treating physicians *Connectivity*: Wi-Fi transmission to a cloud portal for remote review *Median setup time*: 5 min (vs. conv-EEG: 239 min; vs. after-hours conv-EEG: 288 min) *Ease-of-use rating (scale 1–5)*: mean score: 4.7 ± 0.6 *Adverse events*: no major adverse effects reported *Deployment*: successfully deployed across all ICU settings	POC-EEG diagnostic performance (vs. initial clinical suspicion): *Seizure detection*: - Se from 77.8% to 100% - Sp from 63.9% to 89%	POC-EEG clinical impact (after vs. before): Significant change in seizure suspicion (p < 0.0001) (40.2%) - Reduced suspicion for seizures: −32.6% (59 patients) - Increased suspicion for seizures: + 7.3% (13 patients) Modifications in ASM use (20.1%): - Reduced ASM escalation: 12.9% (23 patients) - Initiated ASM escalation: 7.3% (13 patients) Confidence in diagnosis and treatment: significantly increased (*p* < 0.0001)
15.	*First author*: Wright [7], 2021, USA *Sample size*: 38 patients *Setting*: ED, two hospital sites (Community hospital, Academic hospital) *Condition*: suspected NCSE due to various etiologies were identified (e.g., SE, stroke, TBI, toxic-metabolic encephalopathies, and idiopathic AMS) *EEG system*: Ceribell rr-EEG + Brain Stethoscope *EEG Interpretation*: - Community hospital: emergency physician (sonified EEG) + neurologist visual review (reference standard) - Academic hospital: on-call EEG fellow visual review + neurologist visual review (reference standard)	POC-EEG: *EEG setup*: - Community hospital: emergency physicians - Academic hospital: ED technicians *Time from EEG start to diagnosis*: - Academic hospital: median 75 min	POC-EEG Diagnostic Performance: Community hospital site only: Sonified rr-EEG - *Seizure detection*: Se 100%, Sp 92% - *Agreement between emergency physicians and neurologists*: 1/2 seizures diagnosed confirmed (1 NCSE; 1 reclassified as diffuse slowing false positive); 12/12 cases ruled out for seizures Academic hospital site (rr-EEG): - 3 patients (11%) had ongoing seizures or HEP - 1 patient (3%) was in NCSE	Overall impact of POC-EEG across both sites: - Diagnostic confirmation: 42% of cases - Changes in clinical management: 53% of cases - Expedited disposition: 21% of cases
16.	*First author*: Kamousi [33], 2021, USA *Sample size*: 353 rr-EEG recordings *Condition*: Adults with AMS requiring rr-EEG monitoring for suspected seizures *Settings*: ICUs and EDs across six academic and community hospitals *EEG system*: Ceribell monitoring; Clarity machine learning algorithm *Reference standard*: Ceribell review by two independent neurologists	POC-EEG: *Recording duration*: mean 233 min *Mean EEG duration*: 233 ± 227 min *Connectivity*: wirelessly transmitted to a cloud-based system	POC-EEG Diagnostic Performance: *Seizure detection accuracy at different burden thresholds*: - ≥90% SzB (SE alert): Se 100%, Sp 93% - ≥50% seizure burden: Se 100%, Sp 82% - ≥10% seizure burden: Se 88%, Sp 60% Sensitivity for detecting any seizures: 88% NPV: 99% for seizure detection FPR: 0.36 per hour of EEG monitoring FP 24/353 (at ≥90% threshold); most false positives (87.5%) occurred in EEGs labeled as HEP by neurologists *Concordance with Expert Consensus*: - 9/9 Clarity correctly detected SE - 41/ 47 Clarity correctly detected seizure events	Potential POC-EEG application: - SE Alerts (≥90% Seizure Burden) triggered in all 9 SE cases, aiding in timely identification Potential Triage Benefits: - Rapid identification of high-risk patients for urgent intervention and stratification of SzB to optimize neurological consultation needs
17.	*First author*: Kalkach-Aparicio [102], 2024, USA *Sample size*: 240 patients (median age: 64) *Conditions*: Persistent altered AMS, clinical concern for NCS, patients at risk for SE *Setting*: University hospital *EEG system*: Ceribell rr-EEG; interpretation by EEG expert *Comparison*: conv-EEG performed after POC-EEG; interpretation by EEG expert	POC-EEG: *Setup time*: significantly shorter than cEEG (≤5 min vs. 3.5–11 h for cEEG) *EEG setup*: non-specialists	Seizure detection using 2HELPS2B score on rr-EEG vs. cEEG: *AUC for seizure risk prediction*: - rr-EEG: 0.85, *p* = 0.001, noninferior to conv-EEG - conv-EEG (matched cohort): 0.89 (*p* = 0.31, no significant difference) *False-negative rates for 2HELPS2B = 0*: - Rr-EEG 2.1% - Conv-EEG 1.6% (*p* = 1.0) Seizure-relevant EEG patterns in the first hour of rr-EEG were consistent with follow-on cEEG (*p* = 0.95)	Seizure risk prediction: - Patients with low-risk 2HELPS2B scores (0) on rr-EEG had a 2.1% seizure risk over the next 72 h - rr-EEG successfully stratified patients for cEEG allocation, improving access to EEG resources Survival analyses over 72 h: - No significant differences in survival curves between rr-EEG and cEEG groups over a 72 h window
18.	*First author*: Madill [8], 2022, USA *Sample size*: 74 patients (mean age: 61.7 years) *Conditions*: Clinical events concerning seizures (49%), PCA (24%), and unexplained encephalopathy (27%), Settings: ICU and ED, community hospital affiliated with a university hospital *EEG system*: Ceribell rr-EEG; interpretation by on-site neurology and remote epileptologist via tele-EEG *Comparison*: Historical practice before rr-EEG implementation	POC-EEG: *Recording duration*: Up to 12 h per study *After-hours availability:* 69% rr-EEG performed when conv-EEG was unavailable	POC-EEG Diagnostic Performance: *Findings on rr-EEG*: - Seizures: 7 patients (9%) - HEPs: 8 patients (11%) - Slow/normal activity: 59 patients (80%) 90% of seizure detections occurred after hours	Inter-hospital Transfers: Before rr-EEG (historical estimate): 33/74 patients (44.6%) After rr-EEG (pre-tele-EEG phase): 2/40 patients (5.0%) rr-EEG prevented potential transfers: 94% (31/33) Absolute risk reduction: (ARR) of inter-hospital transfers: rr-EEG alone: 39.6% (NNT = 2.5) rr-EEG + tele-EEG: 44.6% (NNT = 2.2) Financial Impact: Estimated cost of inter-hospital transfer: USD 1274 per patient Estimated total savings from prevented transfers: >USD 39,000
19.	*First author*: Kurup [103], 2022, USA *Sample size*: 19 patients *Setting*: ICU *Condition*: Suspected NCS or NCSE *EEG system*: Ceribell rr-EEG *Comparison*: conv-EEG; interpreted by experienced epileptologists	POC-EEG: *Recording duration*: monitoring (conv-EEG: at least 24 h of monitoring after rr-EEG)	EEG Findings: *Seizures detected on both rr-EEG and cEEG (9 patients)*: - High concordance in seizure location, morphology, and rhythmicity between the two methods *Seizures detected only on rr-EEG (6 patients)*: - Most explained by treatment effects (before conv-EEG) *Seizures detected only on conv-EEG(4 patients)*: - Seizures emerged after ASM were tapered - No cases were due to missing midline/parasagittal coverage	
20.	*First author*: Eberhard [5], 2023, USA *Sample size*: 164 EEGs (35 conv-EEGs pre-QI; 115 rr-EEGs post-QI) *Condition*: Suspected seizures *Setting*: Community hospital *EEG system*: Ceribell rr-EEG, real-time sonification and cloud-based *EEG interpretation*: Remote review by on-call neurologist *Comparison* (Reference Standard): Historical control group (pre-QI)	POC-EEG: *EEG setup*: nurses, respiratory therapists, and advanced practice providers *Time-to-EEG initiation*: decreased 7.6-fold (562 min pre-QI vs. 74 min post-QI) *EEG volume*: increased 3.29-fold (35 pre-QI vs. 115 post-QI) *Recording duration*: up to 12 h per patient *Availability*: first-time implementation of 24/7 EEG access in the ED	Seizure Detection Rates (diagnostic yield): *pre-QI*: 0/35 seizures detected *post-QI*: 49/115 (43%) of rr-EEG-identified seizures/HEPs	Patients discharged: ED discharges enabled by rr-EEG: 6 patients Patients discharged within 24 h after rr-EEG: 7 patients LOS Trend toward shorter LOS post-rr-EEG, but not statistically significant: - Median hospital LOS (pre-QI): 7 days - Median hospital LOS (post-QI): 4 days Post-QI Financial Impact Estimated annual savings from reduced LOS: USD 737,818 Additional hospital DRG reimbursements from improved seizure diagnosis: USD 145,580
21.	*First author*: Ward [36], 2023, USA *Sample size*: 88 patients (mean age: 57) *Conditions*: Concern for NCSE (19% exhibited hyperkinetic movements PCA, 46% had a history of seizures and 35% were unresponsive) *Setting*: ICU and ED at a community hospital *EEG system*: - Ceribell rr-EEG system; Clarity-assisted interpretation by critical care fellows - Follow-up standard EEG: Performed the next business day on all patients *Comparison*: Historical control group (patient underwent emergent conv-EEG via inter-hospital transfer with delayed expert interpretation)	POC-EEG: *EEG application*: critical care fellows	POC-EEG Findings: Significant electrographic SzB: 21% - confirmed on follow-up conv-EEG: 4% - required immediate transfer for cEEG: 5% Excluded ESz: 78% FN: 2.4% (ESz on follow-up conv-EEG)	Hospital transfer for emergent EEG: - pre-rr-EEG (2020): 22 pt (mean: 2/month) - post-rr-EEG (2021): 11/88 pt (mean: 1.1/month) Absolute Annualized transfer reduction: 10.8 (*p* = 0.1) Financial impact: - Cost per patient transferred: USD 7500 - Annual cost savings from transfer avoidance: USD 37,401.59 Break-even analysis: - Annual fixed cost of POC-EEG system: USD 119,700.00 - Patient needed to avoid transfer to cover cost (Break-even point): 8.6 per year (0.72 per month) - Observed transfer reduction (0.9 per month) exceeded the break-even threshold
22.	*First author*: Villamar [104], 2023, USA *Sample size*: 21 patients (median age: 64) *Condition*: Comatose PCA patients *Setting*: ICU *EEG system*: Ceribell rr-EEG monitoring as part of routine clinical care; Clarity algorithm (version 4.0) for automated seizure detection *EEG Interpretation*: Board-certified epileptologist retrospective review *Comparison*: - Neurologist-reviewed fm-EEG (20–40 min) performed in selected cases - Retrospective visual review of full raw rr-EEG data by board-certified epileptologists		Raw POC-EEG review findings: *Seizure and SE Detection*: - ESz: 4 of 21 patients (19.0%) - ESE: 2 of 21 patients (9.5%) Performance of Clarity Automated Detection: Clarity system failed to detect any ictal abnormalities	
23.	*First author*: Kozak [6], 2023, USA *Sample size*: 157 adult patients (mean age: 57.7 years) *Conditions*: Clinical suspicion of seizures, unexplained encephalopathy, or PCA *Setting*: ED from a community hospital *EEG system*: Ceribell rr-EEG, Clarity, reviewed by intensivists and neurologists *Comparison*: conv-EEG performed after POC-EEG in 51.6% of cases; interpretation: EEG-trained neurologist (reference standard)	POC-EEG: *EEG application*: emergency physicians and nurses *Door-to-EEG time*: median 1.2 h *Recording duration*: median 2.1 h (monitoring) *After-hours studies*: 55% (63.6% of detecting seizures were performed after hours)	POC-EEG Findings: - Seizures/SE: 14% (SE detected in 6.4% of all patients) - HEPs: 22% - Slowing: 44% - Normal activity: 20%	Treatment changes based on POC-EEG findings: 59.2% of cases POC-EEG findings associated with ASM management changes (*p* < 0.001): - Patients with epileptiform activity (seizures or HEP) had treatment escalation in 52% of cases (vs. 25% in patients with normal/slow EEG, *p* < 0.001) - Patients with normal/slow EEG had treatment de-escalation in 27% of cases (vs. 2% in those with seizures/HEP, *p* < 0.001) LOS: - Door-to-EEG time was positively correlated with longer ED and hospital LOS (*p* < 0.001).
24.	*First author*: Kamousi [35], 2024, USA *Sample size*: 665 POC-EEG recordings *Setting*: 11 hospitals *EEG system*: Ceribell Clarity analysis (two versions tested) *Reference standard*: EEG reviewed post hoc by at least two blinded epileptologists	POC-EEG: *Availability*: Clarity successfully applied to a large multi-hospital dataset	Clarity Diagnostic Performance: - Clarity-detected SE: 36 - 19/20 epileptologist confirmed (95% sensitivity) - 1 FN - 17 FP (16/17 categorized by epileptiform activity; 1 slow/non-epileptiform) Clarity Diagnostic Accuracy for SE detection: - Clarity 6.0: Se 95%; Sp 97%; PPV 53%; NPV 99% - Clarity previous version: Se 95%, Sp 94%, PPV 32%, NPV 98%	
25.	*First author*: Dorriz [34], 2024, USA *Sample size*: 317 POC-EEG recordings *Setting*: U.S. community hospital *EEG system*: Ceribell Clarity outputs (for SE detection); monitoring *Reference standard*: EEG-trained neurologist’s interpretation of POC-EEG recordings	POC-EEG: *Availability*: Clarity successfully applied to POC-EEG recordings in an acute care setting	Clarity concordance with neurologist: Clarity-identified SzB Alerts ≥90%: 10 cases - 6/10 TP (neurologist-confirmed SE cases) - 4/10 FP (still categorized as seizure or HEP) - 0 FN (all neurologist-identified SE were detected by Clarity) Neurologist-categorized SE: 7 cases - 6/7 had SzB 100% - 1/7 had 83.3% SzB; secondary review reclassified as seizure	
26.	*First author*: Desai [105], 2024, USA *Sample size*: 283 patients - POC-EEG cohort: 221 - conv-EEG control: 62 *Conditions*: Acute and non-acute brain injuries *EEG system*: Ceribell rr-EEG *Comparison*: At least 4 h conv-EEG	POC-EEG: *Recording duration*: ≥1 h (vs. ≥4 h for conv-EEG) *Time to acquisition*: faster *Proportion of after-hours EEG*: 66% (vs. conv-EEG 31%; *p* < 0.001) *Time-to-EEG initiation*: reduced *Door-to-EEG time*: median 5.9 h (vs. conv-EEG; median 25.3 h; *p* < 0.0001)		POC-EEG impact on ICU stay: - Median ICU LOS: 4.5 days (vs. conv-EEG: 8.0 days; *p* = 0.011) - Matched cohort analysis confirmed shorter ICU LOS with POC-EEG (3.9 days vs. 8.0 days, *p* = 0.003) - Strongest effect in patients with seizures/HEP (POC-EEG group: 4.8 conv-EEG group: 13.2 day, *p* = 0.03.) Unfavorable Outcomes at discharge (mRS) in POC-EEG group: - Unmatched population: 67% unfavorable mRS (vs. conv-EEG: 76% (*p* = 0.061) - Matched population: 58% unfavorable mRS (*p* = 0.047) Mortality: no significant difference between groups
27.	*First author*: Gururangan [106], 2025, USA *Sample size*: 70 patients (mean age: 75.0 years) *Conditions*: 38 stroke patients (54.3%: 73.7% ischemic, 15.8% hemorrhagic, 10.5% TIA); 32 stroke mimics (45.7%: 46.9% seizures, 28.1% toxic-metabolic encephalopathy, 12.5% hypertensive encephalopathy) *Setting*: Tertiary care community hospital *EEG system*: Ceribell rr-EEG used during stroke codes *Reference standard*: Final stroke vs. stroke mimic diagnosis based on - Neuroimaging findings - Clinical evaluation and stroke team assessment	POC-EEG: *Availability*: successfully implemented as an adjunct to acute stroke evaluation without delaying standard care	POC-EEG findings: *Seizures or HEPs POC-EEG detection*: - 6 (15.8%) stroke patients (2 patients in ESE) - 11 (34.4%) stroke mimic patients (2 patients with expressive aphasia due to focal seizures)	POC-EEG Seizure Detection in Stroke Codes: - Helps confirm or rule out seizures - Aids in differentiating stroke mimics
28.	*First author*: Sheikh [107], 2025, USA *Sample size*: 235 rr-EEG *Setting*: Three hospitals *Condition*: Neurologic conditions with a high risk of seizures *Setting*: ICU or ED *EEG system*: ClarityPro (v 6.0) Setting: Three hospitals *Reference standard*: Expert neurophysiologist consensus review of EEGs	POC-EEG: *Median EEG duration*: 3.6 h *Technical issue*: 29% uninterpretable EEG due to signal degradation and artifact	Performance of Clarity at different SzB thresholds: > 90% SzB: - ESE Detection: Se 43%, Sp 97%, PPV 33% - ESE/pESE Detection: Se 29%, Sp 99%, PPV 78% > 10% SzB: - ESE Detection: Se 86%, Sp 79%, NPV 99%, PPV 11% - ESE/pESE Detection: Se 88%, Sp 84%, NPV 99%, PPV 39%	Clarity application: High specificity (>90%) at higher thresholds (>90%) supports Clarity use in treatment decisions High sensitivity (88%) and NPV (98%) at lower threshold (> 10%) support Clarity use for screening and ruling out ESE

FN: False Negative; FP: False Positive; FPR False Positive Rate; Se Sensibility; Sp Specificity; TP True positive.

## Supplementary Materials

The following supporting information can be downloaded at: https://www.mdpi.com/article/10.3390/neurolint17040048/s1.

## Abbreviations

The following abbreviations are used in this manuscript:

**AIS**	Acute ischemic stroke
**AMS**	Altered mental status
**ASM**	Anti-seizure medication
**BAI**	Brain Abnormality Index
**BD**	Brain death
**BFI**	Brain function index
**BIS**	Bispectral index
**BSEEG**	Bispectral EEG
**BSI**	Brain symmetry index
**CCAT**	Critical Care Air Transport
**CCHR**	Canadian CT Head Rule
**CI**	Concussion Index
**CSs**	Continuous slow waves
**CT+**	Computed Tomography-positive
**CT−**	Computed Tomography-negative
**DA**	Drugs and alcohol
**DAR**	Delta/alpha ratio
**DBATR**	(Delta + Theta)/(Alpha + Beta) Ratio
**DECIDE**	Does Use of Rapid-Response EEG Impact Clinical Decision-Making
**DRG**	Diagnosis-related group
**DSD**	Delirium superimposed on dementia
**DTI**	Diffusion tensor imaging
**EA**	Epileptic activity
**EDs**	Emergency departments
**ESE**	Electrographic SE
**ESz**	Electrographic seizure
**GA**	Genetic algorithm
**GCS**	Glasgow Coma Scale
**GPD**	Generalized PD
**HEP**	Highly epileptiform patterns
**ICU**	Intensive care unit
**IED**	Interictal epileptiform discharge
**LASSO**	Least Absolute Shrinkage and Selection Operator
**LPD**	Lateralized periodic discharges
**LVO**	Large vessel occlusion
**LVO-a**	Anterior LVO
**MTBI-DS**	mTBI discriminant score
**NCS**	Non-convulsive seizure
**NCSE**	Non-convulsive status epilepticus
**NEXUS**	National Emergency X-Radiography Utilization Study
**NOC**	New Orleans Criteria
**NPV**	Negative predictive value
**Non-EA**	Non-epileptic activity
**PABI**	Post-anoxic brain injury
**PD**	Periodic discharges
**POC-EEG**	Point-of-care electroencephalography
**PPV**	Positive predictive value
**QI**	Quality improvement
**RA**	Rhythmic activity
**RDA**	Rhythmic delta activity
**RTP**	Return-to-play
**SAFER-EEG**	Seizure Assessment and Forecasting with Efficient Rapid-EEG
**SBII**	Structural Brain Injury Index
**SE**	Status epilepticus
**SIC**	Structural Injury Classifier
**SRC**	Sports-related concussion
**SVM**	Support Vector Machine
**SW**	Spikes and waves
**SWLDA**	Stepwise Linear Discriminant Analysis
**SzB**	Seizure burden
**TAR**	Theta–alpha ratio
**TBI**	Traumatic brain injury
**UCH-L1**	Ubiquitin C-terminal hydrolase L1
**ViT**	Vision Transformer
**cEEG**	Continuous EEG
**conv-EEG**	Conventional EEG
**c-conv-EEG**	Continuous conventional EEG
**eBFI**	Enhanced BFI
**fm-EEG**	Full-montage EEG
**mTBI**	Mild TBI
**pESE**	Possible ESE
**pdBSI**	Pairwise-derived BSI
**qEEG**	Quantitative EEG
**rEEG**	Routine EEG
**rm-EEG**	Reduced-montage EEG
**rr-EEG**	Rapid-response EEG
**rsBSI**	Revised BSI
