# Supplementary material for: Point-of-Care Electroencephalography in Acute Neurological Care: A Narrative Review
Source: Neurol Int. 2025 Mar 24;17(4):48. doi: 10.3390/neurolint17040048 (PMC12029912; doi:10.3390/neurolint17040048)
Supplement: Supplementary file 1 [file neurolint-17-00048-s001.zip › neurolint-3483127-supplementary.pdf]

Search strategy

Database: Medline
Query: Electroencephalograp*[MeSH] AND (“Point of care Electroencephalograp*" OR “portable Electroencephalograp*” OR “Limited montage Electroencephalograp*" OR “rapid Electroencephalograp*" OR “rapid response Electroencephalograp*" OR “reduced montage Electroencephalograp*" OR “bedside Electroencephalograp*" OR “reduced montage Electroencephalograp*" OR “reduced electrode Electroencephalograp*" OR “reduced lead Electroencephalograp*" OR “minimal montage Electroencephalograp*" OR “reduced channel Electroencephalograp*" OR “abbreviated Electroencephalograp*" OR “brain activity”) AND ("Acute neurological conditions" OR "neurological diagnostics" OR "neurological emergencies" OR "Seizur*" OR "Epilep*" OR "nonconvulsive status epilepticus" OR "Altered mental state" OR "delirium" OR "traumatic brain injury" OR "head injury" OR "brain injury" OR "concussion" OR "stroke" OR "brain ischemia")

Database: Embase
Query: Electroencephalograp* AND ('point of care electroencephalograp*' OR 'rapid electroencephalograp*' OR 'brain activity') AND ('acute neurological conditions' OR 'neurological diagnostics' OR 'neurological emergencies' OR 'seizur*' OR 'epilep*' OR 'nonconvulsive status epilepticus' OR 'traumatic brain injury' OR 'head injury' OR 'brain injury' OR 'concussion' OR 'stroke' OR 'brain ischemia' OR 'delirium')

Database: Scopus
Query: Electroencephalograp* AND (“point of care Electroencephalograp*” OR “rapid Electroencephalograp*” OR “POC Electroencephalograp*” OR “bedside Electroencephalograp*” OR “limited montage Electroencephalograp*” OR “brain monitoring” OR “minimal montage Electroencephalograp*" OR “brain activity”) AND ("Acute neurological conditions" OR "neurological diagnostics" OR "neurological emergencies" OR “seizur*” OR “epilep*” OR “nonconvulsive status epilepticus” OR "traumatic brain injury” OR “head injury” OR “brain injury” OR “concussion” OR "stroke" OR “brain ischemia” OR "delirium")
